# The value of looking ahead: Comparing conventional and strategic Mountain Pine Beetle (*Dendroctonus ponderosae*) management policies in North America

**DOI:** 10.1371/journal.pone.0344860

**Published:** 2026-06-24

**Authors:** Emma J. Hudgins, Rory McIntosh, Mike Undershultz, Chris J. K. MacQuarrie, Devin Goodsman, Denys Yemshanov

**Affiliations:** 1 School of Agriculture, Food and Ecosystem Sciences, The University of Melbourne, Parkville, Victoria, Australia; 2 Forest Service Branch, Saskatchewan Ministry of Environment, Prince Albert, Saskatchewan, Canada; 3 Alberta Forestry and Parks – Forestry Division, Edmonton, Alberta, Canada; 4 Natural Resources Canada, Canadian Forest Service, Great Lakes Forestry Centre, Sault Ste. Marie, Ontario, Canada; 5 Natural Resources Canada, Canadian Forest Service, Northern Forestry Centre, Edmonton, Alberta, Canada; Lavras Federal University, BRAZIL

## Abstract

Forest management in North American montane and boreal biomes is facing multiple challenges, including climate change, wildfires and pest outbreaks. Mountain pine beetle (MPB, *Dendroctonus ponderosae*) is a notable example of a major threat to forest health in western North America whose impacts are compounded by warmer winters, which allowed it to establish further north and east of its historical distributional range. In western Canada, delimiting surveys and control of range-expanding MPB populations are managed through collaborative intergovernmental initiatives. While this approach has been shown to effectively slow MPB spread, these management efforts were planned without assessments of the long-term impacts of survey and control decisions on future rates of MPB spread. Here, we compared the current approach to managing MPB populations driven by recent detections and short-term forecasts against an optimisation-assisted approach that finds a MPB management strategy by factoring in the long-term impacts of survey and management decisions on future spread. We also evaluated a reduced-complexity approach where MPB spread forecasts are factored into decisions without tracking the long-term impacts from future management actions. We found that strategic long-term management could help reduce the future MPB-infested area by 65%−112% compared to the current short-term management strategy. Also, the reduced-complexity approach helped reduce the MPB-invaded area by 16%−77% compared to the conventional short-sighted approach. These results demonstrate the substantial benefits of incorporating longer-term pest spread forecasts and feedback from future management decisions into spatial prioritisations of MPB management efforts.

## Introduction

### Mountain pine beetle epidemic in western Canada

Mountain pine beetle (MPB, *Dendroctonus ponderosae*) is a native pest of pine species in Western North America. Lodgepole pine (*Pinus contorta var. latifolia*) is the dominant host of MPB throughout most of its range in Canada [1], but jack pine (*P. banksiana*), the other widespread pine host of MPB that grows in the boreal forests of Alberta and Saskatchewan, can also support MPB populations, though it may be a less-preferred host [2]*.* The larvae of MPB feed on the phloem of its host trees and the adult stage reproduces in galleries excavated in host tree stems. Mountain pine beetle populations experience occasional epidemics (outbreaks), where populations grow to huge numbers and the insect behaves as an aggressive bark beetle that can attack and kill otherwise healthy host trees. Between epidemics, endemic populations of the insect are small, cannot attack healthy trees, and so subsist in unhealthy and weak trees. The transition between the endemic and epidemic phases is mediated by host tree defences and a pheromone mediated mass attack behaviour [3]. A large number of successful mass attacks in one area can lead to the establishment of an outbreak that, if not controlled, will spread to other nearby trees and forests. Outbreaks only end when the host population is exhausted (i.e., all the trees die) or mass mortality is caused by significant cold weather events [1].

Outbreaks of MPB typically kill vast areas of pine forests and have done so over at least the past 150 years in western North America [4,5]. This has impacted both the ecological function [6] and economic value of forests [7]. During the most recent outbreak in western Canada, MPB killed over 16 million ha of pine forests in British Columbia [8] and, for the first time, breached the northern Rocky Mountains in the Peace Region of northwestern Alberta [9]. Dispersal of MPB populations during the epidemic phase occurs via long-distance spread of tens to several hundreds of kilometers and can be assisted by weather events [10,11]; and by short distance spread within infested stands and local reaggregation mediated by pheromones [1,12,13]. The timely detection and removal of infested trees as soon as they are found is one of the few realistic measures that can limit the growth of local populations and the rate of MPB spread. This method was used to slow the spread of MPB in Canada’s boreal region [14,15].

Significant damages caused by mountain pine beetle negatively impacted the forest industry in western Canada [16] and caused additional carbon emissions from Canada’s forests [17–19]. The incursion of MPB into the Canadian boreal forest biome is a particular threat because the dominant pine species are all susceptible to MPB attack [20,21]. Among these pine species, Jack pine is a particular risk because it occurs throughout the Canadian boreal, and in northern Wisconsin and Michigan, though eastern North America and as far south as New York and New Hampshire [22]. If MPB expands to the eastern boreal forest, this could lead to catastrophic economic damages, reduced water quality, and loss of critical habitat for many threatened species [16]. Therefore, mitigating this invasion and limiting the rate of MPB spread through the Canadian boreal forest has been the focus of significant efforts over the past 20 years.

### Slowing MPB spread

The initial large-scale colonization of Alberta’s boreal biome by MPB occurred following a significant adult beetle dispersal events from British Columbia [9,23].This colonization was followed by a series of successive large- and small-scale dispersal events that allowed MPB populations to invade the boreal forests of central and eastern Alberta [24]. Significant economic and ecological impacts of unmanaged MPB spread led the Government of Alberta to adopt the MPB “Slow-the-Spread” (StS hereafter) program that aimed to promptly detect and remove new infestations of MPB to slow the spread of the insect populations through Alberta’s boreal forest [15]. These efforts included annual aerial surveys focused on the leading edge of the infestation and areas to the west with older, more established populations. Dead and dying pine trees attacked by MPB undergo a characteristic change in the colour of their foliage from green to red. During this process, trees attacked by MPB in the previous year fade from green to yellow or orange. These ‘faders’ can be easily identified during aerial surveys in the fall and winter. Aerial surveys in the Alberta StS program therefore focused on the detection of trees in earlier stages of infestation that were then removed and destroyed in the same winter by ground crews, along with any other attacked trees found nearby [25].

Between 2004 and 2017, Alberta spent ~CAD 500 million on StS efforts for MPB and removed ~1.5 million trees [26]. This resulted in an overall reduction of the MPB population along the leading infestation edge in Alberta of around 41%, and was estimated to have slowed the spread rate by as much as by 60% [27], reduced tree mortality by 79% [28], and yielded annualised benefits of CAD $14 million [29]. While these are promising estimates, the cost-effectiveness of the StS approach has been challenged compared to model-informed approaches [28].

By 2017, MPB populations had invaded the boreal forests of central and eastern Alberta and spread to within 30 km of Alberta’s eastern border with the province of Saskatchewan [24]. The Spread Management Action Collaborative (SMAC) initiative was launched in 2012 between Alberta and Saskatchewan to foster collaborative management of the eastern MPB invasion front [30]. The Government of Saskatchewan contributed almost CAD $4.35 million between 2011–2016 to SMAC [26] and a total of CAD $9.21 million by 2022. The SMAC partners worked to identify priority sites for MPB management by ranking sites for ‘risk of spread’ using three criteria, specifically: 1) The number of infested trees, including estimates of green attacked (undetectable) trees; 2) A modified version of the Stand Susceptibility Index [31] (which is a function of susceptible pine basal area, stand age, and density); and, 3) The spatial connectivity of that stand to other susceptible stands within a 5 km radius.

Notably, ongoing SMAC efforts, while helping reduce the MPB population densities, were not expected to fully stop the spread of MPB, and the infestation was anticipated to approach the Saskatchewan border within the next two decades. The SMAC decision support system used short-term predictions of MPB spread based on current estimated densities or red trees from aerial surveys and the assessments of host tree connectivity and susceptibility near the MPB infestation front. In general, management decisions were implemented in a reactive short-sighted fashion following the outcomes of annual detection surveys. Notably, the SMAC did not seek to predict the outcomes of future management actions on the likelihood of MPB eastward spread. As such, there could have been a more efficient management planning strategy possible in this approach, particularly aiming to slow eastward MPB spread towards Saskatchewan.

### Long-range pest spread forecasts to inform management

Past mathematical modelling efforts of invasive species control indicate that the impacts of management on future invasion dynamics can lead to non-intuitive optimal management strategies [32–34]. Understanding the impacts of long-term (i.e., 10 + years) spread on where and how to manage sites attacked by harmful pests is critical for reducing the overall spread rate of an outbreak. Some sites may contribute disproportionately to long-distance dispersal in ways that are not immediately clear from the estimates based on the current extent of the infestation [35,36]. The consideration of long-term spread alone increases the complexity of a planning exercise compared to spatially implicit models [34] or short-term spatial spread models [33] that have been used to optimise the control of other invaders. Nevertheless, long-term spread models can be fit in a reasonable time horizon using spatial modelling tools, for example, R package *ompr* [37] or MATLAB’s *intlinprog* module [38]. However, considering invasion spread forecasts alone is not sufficient to fully grasp the outcomes of future management decisions on long-term spread. Incorporating these factors can be challenging because the estimation of long-term spread must account for the implications of management decisions in previous time steps on the invader’s population dynamics in the current and future steps [36]. Notably, the full integration of management decisions and spread projections over long planning horizons has high combinatorial complexity that scales up exponentially with the size of the spread area and the length of the planning horizon [39].

In theory, when resources are limited, management should preferentially target the infested sites with the largest anticipated contribution to future long-distance spread of a pest. In the case of MPB, those are infested sites that are likely to produce large numbers of adult beetles that could seed new infestation nuclei elsewhere via long-distance dispersal. In practice, management decisions around MPB in Alberta did not always consider the long-term consequences of MPB spread on the spatial allocation of management actions, nor did they consider how the spatial configuration of past and present management decisions might impede or accelerate future spread.

In this study, we asked how the conventional, reactive MPB management strategy, which was guided by recent detections, compared to a strategy that fully incorporates the spatiotemporal interactions of long-term spread and feedback from future management efforts. We used an existing MPB spread model and SMAC MPB surveillance data as the basis for spatial prioritisation of MPB management efforts across space and time. We also examined the utility of a simpler MPB management approach that considers future MPB spread projections without tracking the impacts of future management actions on long-term spread. In this context, we assessed the effectiveness of a short-sighted approach and explored the benefits of employing more proactive, long-term strategic planning to slow MPB spread, and ultimately delay the occurrence of pine damage from the MPB invasion. We aimed to make a more theoretical contribution in this work, which could provide the foundation for building management prioritisations that can be immediately useable to decision-makers and pest management professionals. This is the first comparison of spatial MPB management strategies to our knowledge, and is the first step in motivating managers to account for long term spread when prioritising management locations for bark beetles.

## Methods

### MPB spread modelling context

We composed a simple model to estimate the long-distance spread of MPB at a coarse spatial scale (5 x 5 km map cells) in the presence and absence of management. Our preference of using a coarse-scale spread model was dictated by the need to incorporate it into a large-scale optimization framework and keep the model tractable enough to be solved in reasonable time. This model was based on historical observations of MPB spread in the presence of management, and empirical estimates of management intensity based on SMAC surveillance data [40]*.* The baseline estimates of current and projected rates of spread at the landscape scale were estimated by dividing historical surveyed MPB range sizes by a management effectiveness term estimated from a finer-scale, more detailed anisotropic spread model fit to the locations of infested trees and stands from annual surveys that also included aspects of MPB’s population dynamics that affect the insect’s detectability and long-distance spread [41]. This model’s estimated management effectiveness is most valid because it covers the same region over the same time period as our model, though we note its estimated effectiveness is also roughly analogous to past estimates of MPB management effectiveness from other studies [42,43], as well as SMAC’s estimated effectiveness [15]. In doing this, we calibrated our simplified spread model such that it recaptured how much farther MPB would spread under present and future conditions in the absence of management. Long-distance dispersal is a critical component of MPB expansion [1,12,13] and so must be always considered in any model. Given the coarse spatial resolution of our model, we assumed that short-distance dispersal would only contribute to local population growth within a 5 × 5-km map cell, and so therefore only attempted to model the establishment of new populations at long distances beyond the 5-km cell size.

### MPB dispersal and growth model

We assumed that an MPB population, after establishing at a given site, would pass through both endemic and epidemic phases. In the endemic phase, population growth occurs, but without notable production of adult beetles (propagules) that could spread over long distances (beyond 5-km) and establish new, viable populations. The epidemic phase is characterized by exponential population growth, where the population produces a sufficient propagule density to sustain long-distance spread to other 5-km map cells.

**Fig 1:**
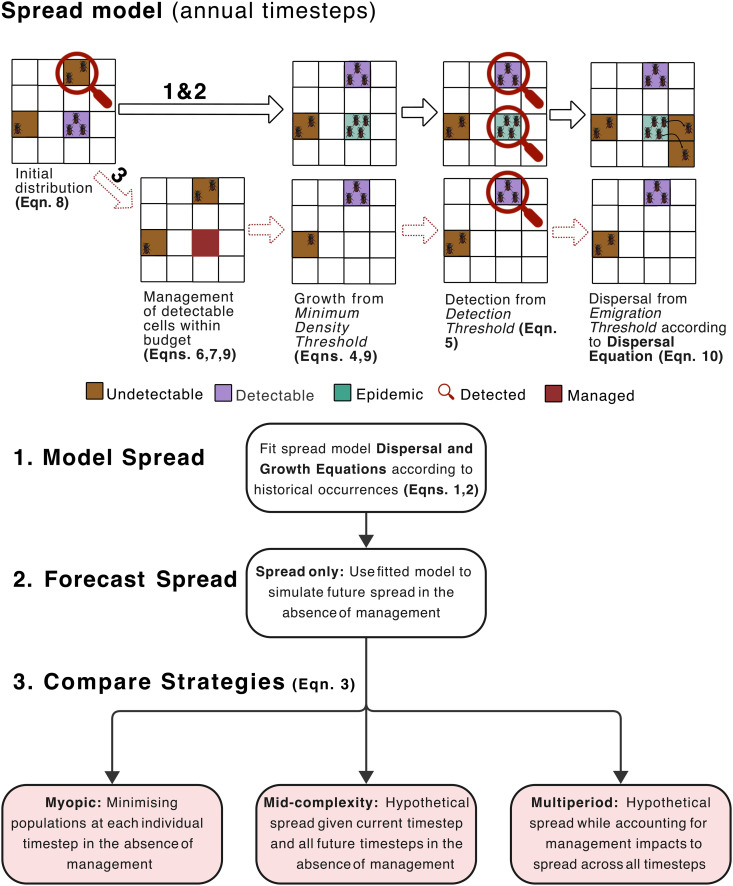
Modelling and optimisation workflow. Numbers in parentheses refer to the model equations. The dashed arrows represent the effect of management actions in the management scenarios listed below (i.e., Myopic, Mid-complexity, and Multiperiod strategies). Italicised text and coloured grid cells reference population dynamics parameters displayed in Fig 2. Beetle icons were created by Eleanor Stern.

We depicted the MPB population dynamics in an infested site with a series of common thresholds that characterize the general population’s behaviour, the capacity to detect the pest and ability to spread propagules to other sites (Fig 1). Once the site *i* gets infested after arrival of MPB propagules from other infested sites *j*, the population density in *i* is set to a small initial value, *w*_*min*_. Without management, the population density in site *i* is expected to grow at an annual rate, *y*_*i*_. Over time, the population eventually achieves a density threshold, *w*_det_, when it can be detected by aerial surveys (i.e., where one or more canopy-level host trees die and turn red within a 5 x 5 km cell)*.*

For some period after initial infestation, the population density in a site is assumed to be insufficient to produce a flow of long-distance propagules that can establish new populations at other sites. However, continued growth at rate *y*_*i*_ eventually allows the population to reach a density level, *w*_spr,_ at which the population in site *i* starts producing propagules that spread over long distances to other sites*.* This threshold defines the beginning of an epidemic phase when the growing population is able to spread propagules to other sites *j*. Eventually, the growing population reaches a carrying capacity, *w*_*max*_ by infesting the majority of host trees in site *j*, and then is expected to collapse. These assumptions replicate the population dynamics of MPB mediated by the mass-attack behaviour and aggregation pheromones [1]. Given the current range of MPB spread rates in Alberta, we assumed that collapse conditions at the broad scale of 5 x 5-km map cells would occur close to or beyond the end of the planning horizon in eastern Alberta, and so the modelling of precise population collapse conditions was not critical in this study. During the epidemic phase, when the population density in site *i* is above the *w*_spr_ level, the pest can spread to surrounding sites within a defined neighbourhood area *N.* We fitted the pattern of dispersal sites around site *j* according to a dispersal equation *e*^*-ar_ij_*^, where *a* is a fitted shape parameter and *r*_*ij*_ is the Euclidean distance between the source and the spread destination sites. We did not need to normalise our dispersal likelihoods to sum to one because the simulated dispersal events depicted a map of randomized binary spread locations rather than a continuous density map of dispersers. Since the populations in newly infested sites were initialized with constant initial population densities *w*_min_, the annual population growth after initial infestation was insensitive to immigration or emigration dynamics.

We generated a set of other sites *j* to which the pest could spread from site *i* in period *t*, by applying the dispersal equation stochastically as a sequence of binomial draws with probabilities $e^{-\alpha r_{ij}}$. This created a stochastic binary pattern of possible spread locations from site *i* in period *t* (Fig S1 in S1 Appendix).

For each site and period *t*, the stochastic simulations generated unique binary dispersal patterns around sites *i*, which could have led to variability in the fine-scale model behaviour. However, due to the law of large numbers, the large number of potential dispersal sites from the surrounding infested sites make this variability negligible when considering the global rate of spread at the scale of the entire landscape. The application of randomized binary dispersal patterns helped incorporate the uncertainty of the long-distance pest spread in an optimization framework where, for each site *i* in time period *t*, the pattern of potential spread locations *j* from *i* was generated exogenously prior to optimization.

We set the maximum radius of the binary dispersal pattern to 40 km based on MPB’s dispersal behaviour [44–46]. While some cases of long-distance MPB spread have been reported to exceed the 40-km distance [1] these events were assumed to be rare. This assumption also constrained the number of other sites that needed to be considered when implementing the long-distance spread from site *i,* and thus helped reduce the size of the optimization problem.

**Fig 2:**
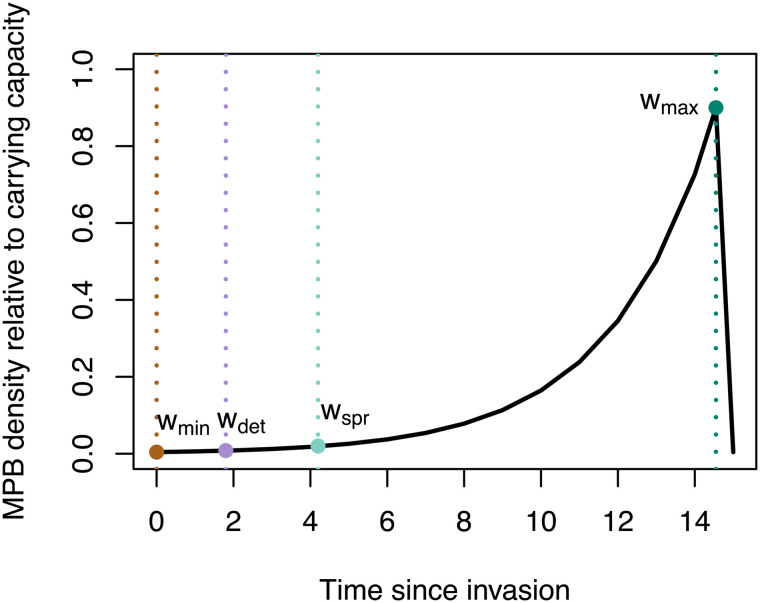
Timing of key mountain pine beetle (MPB) infestation phases. Initial MPB surrogate density *w*_*min*_ = 0.004 is set for newly invaded cells at invasion time *t* = 0. After two years, on average, the population reaches the detectability threshold density *w*_*det*_ = 0.008, and approximately in five years, reaches the epidemic phase threshold, *w*_*spr*_ = 0.02. After being infested for 14-15 years, the population typically collapses when the densities approach *w*_*max*_ = 1.

The population threshold values *w*_min_, *w*_det_ and *w*_spr_ and the annual growth rate depict the temporal dynamics and time lags between initial MPB infestation, the time when the population becomes detectable, and the beginning of the epidemic phase when long-distance spread occurs (Fig 2). In this context, the density values are not depicting the actual population densities in the infested sites *per se* but instead serve as surrogates to recreate the typical timeline of the infestation phases (i.e., when the population becomes detectable and when it enters an epidemic spread phase). All densities in sites *i* were modelled as proportions of the carrying capacity (which was set equal to one). We set the minimum proportional density of MPB in a site once infested, *w*_*min*_*,* to 0.004, which was half the value of the level of proportional density associated with the detection of a MPB infestation at a site via aerial surveys of red trees, so *w*_*det*_ = 0.008. The proportional density when the population enters an epidemic spread phase and starts producing propagules which spread to other sites, *w*_*spr*_, was set to 0.02. The baseline growth rate *y* was set to 1.45 *yr*^*-1*^. The combination of these parameters was designed to produce an infestation timeline where the pest can be detected after at least one year after initial infestation, enters the epidemic (spread) phase, on average, after five years, and reaches carrying capacity over eight to ten years after the beginning of the epidemic phase. At the relatively coarse scale of the 5 x 5 km sites, these values aimed to replicate typical dynamics of MPB infestation phases in Western Canada in the absence of management.

The timing of when a population may become detectable and start producing long-distance propagules depends on the availability and the density of the host trees in a forest site [47,48]. The model thus required information about the variation in host pine densities across the study area. We derived pine densities estimates from the Canadian National Forest Inventory (NFI) dataset [49]. We fitted two additional parameters that allowed the baseline growth rate *y*_*i*_ and the dispersal density threshold *w*_spr_ to vary by ±30% depending on the relative host density in a forest site. The chosen growth rate and the *w*_min_ value were selected to ensure that an infested site reached the maximum pest density within the planning horizon of 11–15 years, or approximately the average length of an MPB outbreak in western Canada [1,4]. Note that during an outbreak in MPB’s native range, the time to achieve *w*_*max*_ will likely be faster than the timespan shown in Fig 2.

We initialized the dispersal model with the map of the 2023 MPB infestation. The pest densities in previously infested sites behind the leading population front (i.e., those not recorded for the first time that year) were assigned relatively high value depicting mature populations capable of dispersing propagules over long distances (i.e., *w*_*spr*_*y*^*t=3*^ = 0.061). This was done to simulate the likely propagule pressure from infested sites in parts of the invaded range where MPB was only subject to sporadic management. The sites along the leading edge of the infestation were set to the threshold for the beginning of the epidemic phase, *w*_*spr*_, which ensured that these sites can spread propagules into the surrounding uninfested area. Sites detected in the past year were initialised to one period of growth above the detection threshold. The simulations were carried for an 11-year planning horizon (2024–2034). We used the R function optim (R Core Team [37]) to determine the most likely values of the dispersal parameters *a* and *N* such that we minimised the mean squared difference in predicted detectable spread area (number of infested cells times the size of each cell) and estimated spread area in the absence of management estimated from Goodsman et al. [41].

### Dispersal equation

For each site *i*, at each time step *t*, we generated a binary dispersal pattern *D*_*ijt*_ using the following best-fitting dispersal equation between sites *i* and *j*:


$$\begin{array}{c} D_{ij}\sim binomial\left(e^{-\text{1}.\text{1}\text{2}r_{ij}}\right),{\text{0}<\;r}_{ij}<\text{8} \end{array}$$

$$\begin{array}{c} D_{ij}=\text{0},{\;r}_{ij}>\text{8} \end{array}$$ (1)

where *r* is the Euclidean distance (divided by 5-km map cell units) between the centroids of 5 × 5 km sites *i* and *j*, such that term *r* is expressed in map cell-width equivalents, and only takes nonzero values at distances below 40 km (8 cell). We also estimated the dispersal threshold *w*_spr_ as a linear function of the relative host volume in site *i*, *h*_*i*_, i.e.,:


$$\begin{array}{c} w_{{spr}_{i}}=\text{0}.\text{0}\text{2}+\text{0}.\text{0}\text{1}\text{6}h_{i},\;\text{0}.\text{0014}<w_{{spr}_{i}}<\text{0}.\text{026} \end{array}$$ (2)

For each site *i*, the *h*_*i*_ values were extracted from the NFI [49]. We also adjusted the relative growth rate *y*_*i*_ by the host relative volume in site *i*, assuming that propagules are more likely to spread from sites with higher volumes of suitable hosts (i.e., these sites were assumed to contain more productive trees), i.e.,:


$$\begin{array}{c} y_{i}=\text{1}.\text{4}\text{5}+\text{0}.\text{4}\text{9}h_{i},\;\text{1}.\text{015}<y_{i}<\;\text{1}.\text{885} \end{array}$$ (3)

The upper and lower bounds on the range of *w*_spr *i*_ and *y*_*i*_ values across the study area ensured that the spread behaviour in sites *i* deviated no more than 30% from their mean values. This range aimed to keep the spread rates consistent with the variation of historical invasion patterns in Alberta [14]. This model had a root mean squared error in observed vs. predicted spread area of 79.3 cells (1982.5 km^2^, R^2^ = 0.55).

### Management strategies

Our optimisation model included both the impacts of future MPB spread and the feedback from management actions (infested tree removal) on MPB population density (Fig 1). We tested three management strategies that assumed different levels of planning foresight and abilities to model the impact of management interventions on future MPB spread:

The “**single-period” (myopic) model** depicted a one-year horizon, reactive management approach where tree removals closely followed the outcomes of annual aerial tree surveys. This model allocated management actions (i.e., tree removals) within a given year based on the detections made in that year and previous years. The removal of infested trees targeted the sites with the greatest estimated capacity to reduce the current size of MPB’s established range and host exposure over the current period but without estimating the impacts of these actions on future spread. This model reflects the management approach used by the SMAC, which estimated the dispersal capacity at a given site under conditions in the current and previous year. Note, however, that our model is slightly simplified version of the SMAC approach, as it does not consider the impact of tree removals in the current year on any future rates of MPB spread. The SMAC program incorporated a consideration of MPB spread over short time frames (i.e., 1–3 years) as part of its decision making. This model has the lowest computational complexity.

The “**single period with spread” model** examined a medium-term horizon where tree removals at the current timestep might impact future spread. This model, like the myopic model, did not consider the spatial feedback from future management actions on future spread beyond the current period, however, it included projections of future spread without feedback from management and minimized the spread area over the whole planning horizon. Forecasting future spread without feedback from future management actions is a simple computational task for which several tools are available.

The “**multiperiod” (full) model** examined all combinations of future spread and management across the entire planning time horizon and how they would impact future rate of spread. Tree removals were planned for all periods *t* = 1,…,*T* and the optimization model tracked their impacts on future spread over the entire planning horizon *T*. This allowed the model to find those strategic locations where tree removal could disproportionately reduce the capacity for future long-distance MPB spread.

The multiperiod planning problem is the most computationally complex of the three management models. The single-period model with spread is a mid-complexity solution and the single-period myopic model is the simplest of the three models. Hereafter, we refer to these three models as the full, mid-complexity and myopic models, respectively. We applied these models to assess how much improvement the full problem solution could provide over the simpler, mid-complexity model solution, and under which scenarios all the three models would diverge or converge. A comparison of the different models allows us to evaluate the relative advantages of these different approaches, should they be applied to similar management problems.

### Problem formulation for multiperiod model

Below, we depict a non-linearized model formulation that includes the products of decision variables. The full formulation is provided in S2 Appendix and a description of all symbols used is provided in Table S1 in S2 Appendix. Fig 1 shows how each of these equations relate to our overall workflow.

Our pest management problem minimizes the total number of sites *i* with the detected infestations over the planning horizon of |*T|* periods*,* i.e.,:


$$\begin{array}{c} \text{min}\sum_{i=\text{1}}^{I}\sum_{t=\text{1}}^{T}v_{it}+fu_{it} \end{array}$$ (4)

Objective function (4) sums the number of detected infested sites *v*_*it*_ and includes a small penalty that denotes the number of infested undetected sites $u_{it}$. This penalty helps to break symmetry and avoid ties by slightly adjusting the objective value via tracking the number of the infested undetected sites multiplied by a very small scaling factor, *f =* 0.001.

The non-negative variable *w*_*it*_ defines the relative population density in site *i* in period *t*. The binary decision variable, *u*_*it*_*,* defines that site *i* is infested in period *t* (*u*_*it*_ = 1 and *u*_*it*_ = 0 otherwise) The site *i* is considered infested if it has a pest population density *w*_*it*_ above the minimum density threshold *w*_*min*_*,* i.e.,:


$$\begin{array}{c} u_{it}=\left\{ \;\;\;\begin{array}{c} \text{1},\;w_{it}\geq \;w_{min}\\ \text{0},\;w_{it}<w_{min}\; \end{array}\right.\; \end{array}$$ (5)

Site *i* is considered detectable (defined by the binary decision variable *v*_*it*_ = 1) if it has a population density above the detection threshold, *w*_*det*_*.* In other words;


$$\begin{array}{c} v_{it}=\left\{ \;\;\;\begin{array}{c} \text{1},\;w_{it}\geq \;w_{det}\;\\ \text{0},\;w_{it}\;<w_{det} \end{array}\right.\; \end{array}$$ (6)

The binary decision variable *q*_*it*_ defines that site *i* has been managed in period *t* (*q*_*it*_ = 1 and *q*_*it*_ = 0 otherwise). Only detectable infestations can be managed (7).


$$\begin{array}{c} q_{it}=\text{0},\;w_{it}\leq w_{det},\;w_{it}\;\geq w_{imax} \end{array}$$ (7)

The total number of sites that can be managed (i.e., where the infested trees can be removed) in period *t* is limited by an upper bound representing the treatment budget *B* (8)*.*


$$\begin{array}{c} \sum_{i=\text{1}}^{I}q_{it}{(c_{fix}+c_{var}w^{\prime }}_{it})\;\leq B\; \end{array}$$ (8)

The cost of managing site *i* in period *t* is a function of the population density in *i* after the dispersal and growth phase, *w’*_*it*_. The cost of managing site *i* includes a fixed cost portion, *c*_*fix*_, of transporting a management crew to site *i* where they complete the follow up inspections, and a variable cost component, *c*_var_, which depends on the relative density of the detected MPB population in site *i* and thus the number of infested trees which may need to be inspected and removed by the management crew. This implicitly assumes that MPB density is proportional to the number of trees to be scheduled for removal. Removals are done by felling each infested tree with a chainsaw and then burning the tree at the site, which results in the complete destruction of all propagules in the tree. All tree removals are completed in the winter months when MPB is in a quiescent state and unable to escape treatment, and wildfire hazard is low.

The model also required the initial values for the population densities in sites *i*. We initialised the population densities from the estimates based on dispersal and growth equations in the absence of management, $w_{iinit}$, i.e.,:


$$\begin{array}{c} w_{it}\;=\;w_{iinit},\;t=\text{1} \end{array}$$ (9)

The infested population, once established in site *i*, is expected to grow at an annual rate, $y_{i}$ until it reaches the carrying capacity, $w_{imax}$. Management reduces the $w_{it}$ value by the management factor *q*_*it*_*e*, where parameter *e* defines the management effectiveness (10).


$$\begin{array}{c} \overset{\prime }{\underset{it}{w}}\;=\;y_{i}w_{it}(\text{1}\;-\;q_{it}e)\;,\;\text{0}<=\overset{\prime }{\underset{it}{w}}<=w_{imax} \end{array}$$ (10)

Once the relative population density at a site has reached the dispersal threshold, *w*_*spr*_*,* in period *t*, its propagules can spread from *i* elsewhere. The spread from *i* in period *t* proceeds according to an empirically estimated randomized binary pattern of sites *j* around site *i* based on an exponential dispersal equation. This pattern is defined by the binary parameter $D_{jit}$*,* which indicates the sites *i* across the study area where propagules could spread from site *j* after the population in *j* has reached the dispersal threshold, *w*_*spr*_, in period *t* ($D_{jit}$ = 1 and $D_{jit}$ = 0 otherwise) (see Fig S1 in S1 Appendix for a binary spread pattern example). For each site *i* in period *t*, the distribution of the randomized binary patterns of $D_{jit}$values was generated with the dispersal model that we fitted externally using the spatial spread model and the *optim* package [37]*.* When the population density, $\overset{\prime }{\underset{it}{w}}$*,* in site *j* has reached or exceeded the dispersal threshold, *w*_spr,_ in period *t,* the sites *i* around *j* with the $D_{jit}$ values equal to one were considered receiving propagules from *j* and assigned the minimum population density level, *w*_min_, in period *t*. The equation that accounts for both dispersal after growth and treatment is thus represented by:


$$\begin{array}{c} w_{i(t+\text{1})}=\left\{ \;\;\;\begin{array}{cc} w_{min}\underset{\;j\in \overset{\prime }{\underset{jt}{\;w}}\geq w_{spr}\;}{\text{max}}\left[D_{jit}\right], & \overset{\prime }{\underset{it}{w}}=\text{0}\\ {w^{\prime }}_{it}, & \text{0}<=\overset{\prime }{\underset{it}{w}}<=w_{max}\\ w_{max\;}, & \overset{\prime }{\underset{it}{w}}\geq \;w_{max} \end{array}\right.\; \end{array}$$ (11)

We assumed that long-distance spread11is only possible from sites with population densities above the spread threshold, $w_{spr}$, and below the carrying capacity limit, $w_{i\;max\;}$*.* Above the minimum density ($w_{min}$), the population growth is governed only by the previous timestep’s population size, $w_{it}$*,* the growth rate, $y_{i}$ and the management actions*.*

We set the management efficiency *e* to 0.5, the management budget *B* to 150 units, with a fixed unit cost, *c*_*fix*_, of 0.7 and a variable unit cost of *c*_*var*_ = 20. The management efficiency and the unit cost values were chosen through a series of tests to recreate the approximate MPB spread rates in the scenarios with and without management according to management effectiveness estimates from Goodsman et al. [41]. Given the coarse spatial resolution of the study (5 × 5 km), we did not assume the cost savings would accrue from managing multiple grid cells located near one another. Instead, we treated each site’s management action as a separate trip, making the site management costs independent of the spatial arrangement of management. Based on recent history of site treatments across Alberta, we did not impose a travel time limit on the selection of the managed sites.

We developed the full model first and then simplified the code to formulate the myopic and mid-complexity models. This helped minimise the inconsistencies across the models. Below we list the modifications of the full model which were required to formulate the myopic and mid-complexity models.

### Myopic model

Our myopic model is a sequence of single timestep models which, at each annual time step *t,* minimize the total number of infested sites while applying management only at a given time step. To generate the solution for the whole time horizon *T*, the myopic model was solved in sequence at each timestep *t,* with the solution at the previous step *t*-1 serving as an input for the model at time step *t*. Programmatically, the first set of management decisions in period *t =* 1 was chosen by optimising the objective function (4), subject to all constraints restricted to period *t =* 1. To allocate management decisions in period *t =* 2*,* the decision variables that defined the treatment and infestation status of sites *i* in period *t =* 1 were fixed*,* and the objective function was optimised only for period *t =* 2 and so on in sequence until reaching the end of the planning horizon, *t* = |*T|*.

### Mid-complexity model

The mid-complexity model was also implemented as a sequence of |*T|* separate minimizations. However, unlike the myopic single-period model, the mid-complexity model projected the future spread over the entire horizon *T* similarly to the full model. The mid-complexity model allocated treatment decisions for the current time step *t* only*,* similarly to the myopic model, but also forecasted the future spread over horizon *T* without feedback from management actions. In this way, the future spread forecast only included the feedback from the management actions done in the current and previous periods. For instance, when calculating the management decisions for *t =* 1, management was only allowed in *t =* 1*,* and management decision variables were set to zero for all future time steps 2,…,|*T*|. As in the myopic model, when calculating decisions at timestep *t =* 2*,* all prior management and binary indicator decision variables were fixed and could not be changed. However, the future spread was estimated for the entire planning horizon 2,…,|*T*|.

### Sensitivity analysis

Model construction required defining relative approximate values for several parameters. Since our aim was to compare the general impact of strategic MPB management vs. the short-sighted, reactive strategy at a very coarse scale, the precise estimation of fine-scale MPB dynamics was not critical. Instead, our parameters were chosen to roughly recapture the timelines and approximate spread rates at the whole-landscape level and depict the appropriate lag times between infestation, detection, subsequent spread and population collapse phases. Nevertheless, we examined a range of sensitivity scenarios to assess the impact of changing the model parameter values on the variability of spatial management priorities. This included adjusting the maximum spread distance, the key population density thresholds *w*_*min*_ and *w*_*det,*_ the management efficiency *e*, the management unit cost, the overall management budget level, and the combination of the available treatment budget vs. the capacity to detect the infestation. We evaluated the response of the MPB spread rate to changes in each model parameter value, one parameter at a time (see Table 1). We also tested how sensitive the management scenario outcomes were to the changes in the size of the tree removal budget.

**Table 1:** Parameter values used across sensitivity analyses, mgmt = management. Model parameter changes relative to the baseline scenario (best estimates of current dynamics) values are bolded.

Sensitivity analysis	Budget *B*	Fixed mgmt cost portion, *c*_*fix*_	Variable mgmt cost portion, c_var_	Mgmt efficiency, *e*	Initial population density, *w*_*min*_	Detection density threshold, *w*_*det*_	Spread density threshold, *w*_*spr*_	Annual growth rate, *y*
Base	150	0.7	20	0.5	0.004	0.008	0.022	1.65
Increase management efficiency, *e* by 30%	150	0.7	20	**0.65**	0.004	0.008	0.022	1.65
Decrease detection threshold, ***w***_***det***_ by 100%	150	0.7	20	0.65	0.004	**0.004**	0.022	1.65
Increase detection threshold, ***w***_***det***_ by 100%	150	0.7	20	0.65	0.004	**0.012**	0.022	1.65
Reduce minimum pest density, ***w***_***min***_ by 50%	150	0.7	20	0.65	**0.002**	0.008	0.022	1.65
Increase minimum pest density, ***w***_***min***_ by 100%	150	0.7	20	0.65	**0.008**	0.008	0.022	1.65
Increase budget, *B*, by 100%	**300**	0.7	20	0.5	0.004	0.008	0.022	1.65
Higher fixed cost portion for management, *c*_*fix*_	150	**0.8**	**20**	0.5	0.004	0.008	0.022	1.65
Lower fixed management cost, *c*_*fix*_ by 14% and increase variable cost, *c*_*var*_ by 25%	150	**0.6**	**25**	0.5	0.004	0.008	0.022	1.65
Decrease budget, *B*, by 17%	**125**	0.7	20	0.5	0.004	0.008	0.022	1.65
Decrease management efficiency, *e,* by 25%	150	0.7	20	**0.45**	0.004	0.008	0.022	1.65
Decrease budget, *B,* by 50%	**75**	0.7	20	0.5	0.004	0.008	0.022	1.65
Decrease budget, *B,* by 50% & increase detection threshold, ***w***_***det,***_ by 100%	**75**	0.7	20	0.5	0.004	**0.012**	0.022	1.65

We composed the models in the GAMS environment [50], initially working on Natural Resources Canada and UniMelb workstations for debugging. We solved all models with the GUROBI linear programming solver [51] on a Linux virtual machine with 800 GB RAM and 96 threads at high performance Spartan Computing Cluster at University of Melbourne. The myopic model had one-step formulation and was solved within a few minutes. The mid-complexity model solved in 20–40 s per time step, leading to solution times of 7–8 min in the baseline scenario (best estimate of current dynamics). The full model was warm started with the myopic model solution and solved for 36 h or until reaching the 0.1% optimality gap.

## Results

### Infestation patterns

The solver achieved a 0.55–2.3% optimality gap after 36 h across scenarios for the full model, with larger gap values in the solutions with higher treatment effectiveness and higher budget levels. The mid-complexity and myopic models converged to near-optimality consistently across all scenarios.

**Fig 3:**
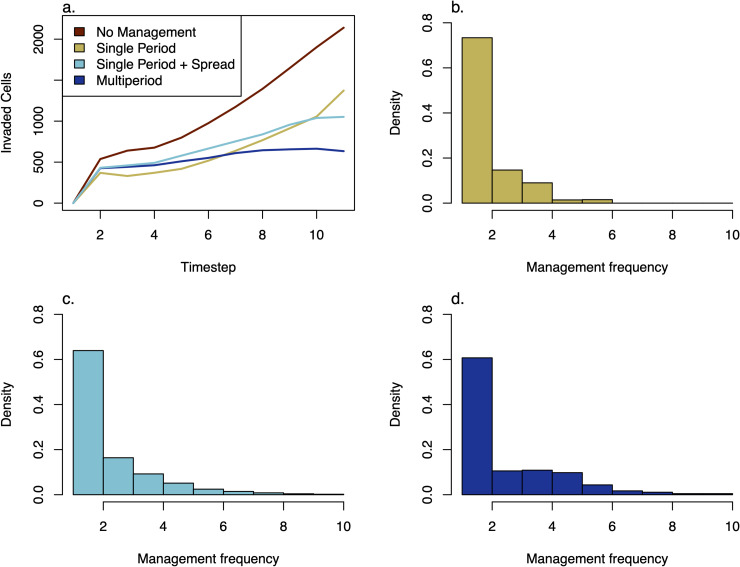
Baseline scenario solutions (based on the best estimate of current MPB. **a)** the number of invaded 5x5-km sites across each model scenario; **b-d)** the frequency of re-treatments of an infested site over planning horizon *T* in each model scenario.

Our simulations of MPB spread pattern without management revealed that spread starts as exponential but later stabilizes at a constant linear spread rate as a travelling wave. All the myopic, mid-complexity and full model scenarios were able to decrease the spread rates but to different extents. In the myopic solutions, tree removals slowed the linear spread rates, but the infested area kept increasing exponentially (Fig 3a and Table 1). In the mid-complexity solutions, spread rates tended to slow towards the end of the planning horizon. The solutions to the full model yielded the greatest spread rate reductions. The mid-complexity and full models were not as effective as the myopic model at reducing the spread rate at the beginning of the planning horizon. This behaviour is expected because, unlike in the myopic model, the management decisions in the mid-complexity and full models aimed to strategically reduce the spread rate over the entire planning horizon and were not as efficient as the myopic model in the first time periods. The full models, though, resulted in the greatest reduction of eastward MPB spread, with a net 120-km reduction in eastward spread relative to the no-treatment scenario, compared to a net 0 km of reduction in eastward spread for the myopic model solution, and a net 100 km of spread reduction for the mid-complexity model over the planning period (Fig S2 in S1 Appendix).

Our results revealed notable differences among the model scenarios in the allocation of re-occurring management actions. The myopic model largely applied the same strategy across the infested area. This strategy aimed to manage the detected infested sites at low population densities just above the detection threshold, *w*_det_, as soon after the detection as possible, and rarely applied repeated treatments to the same sites at later time steps (Fig 3b and Table 2). This strategy was characterized by a low variation in the population densities in infested sites at the time of treatment (Table 3).

**Table 2:** The objective function values and the proportion of the detected infested area relative to the no-management scenario (see Table 1 for scenario descriptions).

Changes in the model parameters (sensitivity analysis)	Objective function value				Infested area proportion vs. the no-management scenario		
	No management	Single-period myopic model	Mid-complexity model	Multiperiod full model	Single-period myopic model	Mid-complexity model	Multiperiod full model
Baseline scenario	2327.28	1534.67	1054.11	657.56	0.62	0.39	0.14
Increase management efficiency, *e* by 30%	2327.28	1248.44	673.99	454.95	0.36	0.15	0.03
Decrease detection threshold, ***w***_***det***_ by 100%	2714.28	2070.95	1359.78	875.21	0.66	0.40	0.12
Increase detection threshold, ***w***_***det***_ by 100%	2068.28	1439.79	852.22	593.45	0.63	0.36	0.21
Reduce minimum pest density, ***w***_***min***_ by 50%	3739.42	2990.17	1427.02	1102.7	0.76	0.30	0.18
Increase minimum pest density, ***w***_***min***_ by 100%	1608.77	919.04	858.11	497.11	0.51	0.46	0.12
Increase budget, *B*, by 100%	2327.28	633.58	819.01	210.38	0.23	0.30	−0.12
Higher fixed cost portion for management, *c*_*fix*_	2327.28	1625.75	1076.17	721.34	0.67	0.39	0.18
Lower fixed management cost, *c*_*fix*_ by 14% and increase variable cost, *c*_*var*_ by 25%	2327.28	1493.65	1050.08	623.79	0.61	0.39	0.13
Decrease budget, *B*, by 17%	2327.28	1684.80	1093.17	715.39	0.71	0.51	0.33
Decrease management efficiency, *e,* by 25%	2327.28	1632.74	1192.23	718.63	0.69	0.47	0.17
Decrease budget, *B,* by 50%	2327.28	1959.00	1259.27	971.52	0.84	0.48	0.31
Decrease budget, *B,* by 50% and increase detection threshold, ***w***_***det,***_ by 100%	2068.28	1730.04	1084.27	845.11	0.83	0.49	0.35

**Table 3:** Proportion of sites managed immediately after detection (columns 1-3) vs. in later planning periods (columns 4-6).

	Manage immediately after detection			Manage at later time steps		
Changes in the model parameter (sensitivity analysis)	Single-period myopic model	Mid-complexity model	Multiperiod full model	Single-period myopic model	Mid-complexity model	Multiperiod full model
Baseline scenario	0.97	0.84	0.82	0.03	0.09	0.12
Increase management efficiency, *e* by 30%	**0.95** ^**a**^	**0.71**	**0.71**	0.03	0.17	0.17
Decrease detection threshold, ***w***_***det***_ by 100%	**0.11**	**0.46**	**0.39**	0.85	0.5	0.51
Increase detection threshold, ***w***_***det***_ by 100%	**0.07**	**0.57**	**0.49**	0.13	0.24	0.33
Reduce minimum pest density, ***w***_***min***_ by 50%	**0.94**	**0.59**	**0.66**	0.03	0.21	0.20
Increase minimum pest density, ***w***_***min***_ by 100%	**0.97**	**0.81**	**0.77**	0.02	0.08	0.14
Increase budget, *B*, by 100%	0.92	0.88	0.66	0.08	0.07	0.18
Higher fixed cost portion for management, *c*_*fix*_	0.97	0.81	0.79	0.03	0.10	0.14
Lower fixed management cost, *c*_*fix*_ by 14% and increase variable cost, *c*_*var*_ by 25%	0.98	0.85	0.77	0.02	0.08	0.15
Decrease budget, *B*, by 17%	0.98	0.80	0.80	0.02	0.09	0.14
Decrease management efficiency, *e,* by 25%	0.99	0.82	0.76	0.01	0.11	0.17
Decrease budget, *B,* by 50%	0.99	0.73	0.76	0.01	0.12	0.15
Decrease budget, *B,* by 50% & increase detection threshold, ***w***_***det,***_ by 100%	**0.12**	**0.42**	**0.40**	0.88	0.48	0.53

^a^Large differences in the number of sites managed at each lag after detection across the sensitivity analyses are shown in bold.

**Fig 4:**
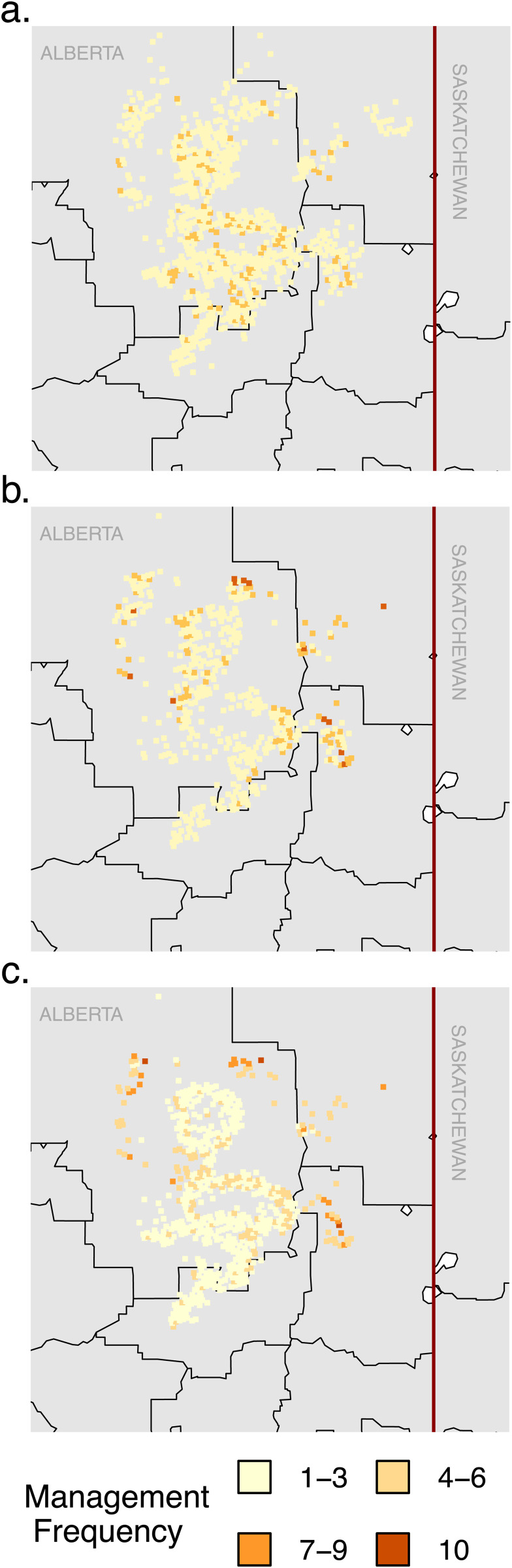
The frequency of re-treatments of the infested sites over the planning horizon for the single period (myopic), mid-complexity and full model solutions. The Alberta-Saskatchewan border is delineated by a dark red line. Each square depicts a 5 × 5 km map cell. Basemap contains information from Statistics Canada, licensed under Open Government Licence – Canada, from R package canadamaps v2.0.0 [52].

In contrast, the mid-complexity and full models tended to manage sites near the leading edge of the infestation, and many sites near the leading edge were re-treated several times over the planning horizon (Fig 4). The heavier tail of the distribution of the management frequencies of the infested sites in Fig 2d indicates that some sites were managed nearly every year, or every second year. The mid-complexity model solutions also produced a distribution of treatment frequencies with a fatter tail which indicates that many sites were treated multiple times over the planning horizon *T* (Fig 3c). This model tended to re-treat the infested sites that contributed the most to the long-distance spread. However, these repeated tree removal efforts were not as focused on the same sites as the multiple treatments prescribed by the full model. This likely occurred because the full model was able to track the feedback from future management decisions on the rate of spread over the entire planning horizon.

**Fig 5:**
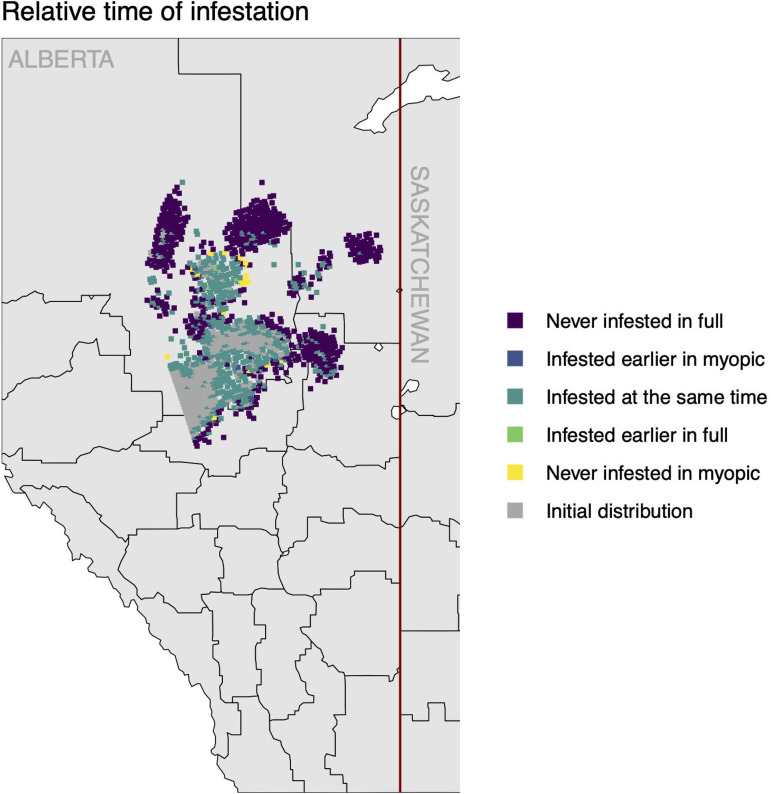
Differences in infestation times between the myopic and full model solutions. The invaded area at *t =* 1 is shown in grey. Basemap contains information from Statistics Canada, licensed under Open Government Licence – Canada, from R package canadamaps v2.0.0 [52].

The timing of infestations and the strategic placement of treatments helped reduce the future spread rate, so that sites that were infested in the myopic model werenever infested in the full model (purple cells in Fig 5). The full model was able to prevent many potential new long-distance infestations (termed remote nuclei in Shigesada et al. [53]) by sacrificing the treatment of the infested sites in the core of the invaded area (shown in yellow in Fig 4). This strategic allocation of treatments helped reduce the number of sites where population densities were sufficient to produce long-distance spread, which also reduced the creation of new infestation nuclei via long-distance spread.

Overall, the full model yielded a greater reduction in MPB spread area than the mid-complexity and myopic models (Table 2). Even in the scenario where MPB populations could establish at very low densities, detection was only possible at very high densities, and with long time delays, the mid-complexity scenario was able to reduce the infested area by 66%, and the full model yielded a further 15% reduction over the mid-complexity scenario. Only when the budget level got unrealistically high and sufficient to treat all detected high-risk locations was the myopic model able to outperform the mid-complexity approach.

Increasing the capacity to detect new infestations did not reduce the infested area in the mid-complexity or full models. Increasing the effectiveness of management efforts, however, slightly decreased the performance of the mid-complexity model compared to the myopic model. However, this also improved the performance of the full model and caused a large reduction of the infested area in the model solutions. The invasion trajectories in the mid-complexity and full model solutions became more similar when the effectiveness of treatments increased.

Reducing the management budget altered the spatial strategy of the full model, which tended to re-treat the same sites less frequently (Fig 4, right panel). However, this change did not impact the effectiveness of the treatments in the full model compared to the myopic model (Table 2). Also, more frequent treatments of the same sites were observed in the scenarios with poorer detectability (when the infestations could only be detected at a later development stage and, subsequently, the management efforts could be applied at higher densities closer or above the epidemic spread threshold *w*_spr_, Table 3). In these scenarios, more frequent treatments were allocated to those sites with population densities that were just above the detection threshold, and fewer sites were treated multiple times (Table 4). This indicates that an increased lag between the actual infestation and the time of detection is likely to impede the strategic placement of treatments efforts. Even in the scenarios with higher management effectiveness, the full model eventually gave up on managing the infested sites near the core of the infestation (sites in yellow in Fig S4 in S3 Appendix). We also observed that a larger number of sites were infested much later in the myopic model relative to the full model in high efficiency scenarios (marked in yellow in the infested core areas in Figs S6, S8 and S10 in S3 Appendix). This indicates that the myopic model can delay the infestation in a core area if the efficacy of the management is sufficiently high. The myopic model was able to suppress the creation of new infestation nuclei, but only in the large-budget solutions which allowed the treatment of most of the detected infested sites (Figs S13 and S14 in S3 Appendix).

**Table 4:** Mean population densities (*w*_*it*_) in managed sites *i* over planning horizon *T*, and the number of times a site with the detected infestation was re-treated over planning horizon *T*, ($\sum_{t}{\overset{―}{q}}_{it})$.

	Population density			Site Management Frequency		
Changes in the model parameter (sensitivity analysis)	Single-period myopic model	Mid-complexity model	Multiperiod full model	Single-period myopic model	Mid-complexity model	Multiperiod full model
Baseline scenario	0.0091 ± 0.78	0.0099 ± 0.54	0.0097 ± 0.22	2.01 ± 1.14	2.31 ± 1.66	2.67 ± 1.83
Increase management efficiency, *e* by 30%	0.0093 ± 0.94	0.0201 ± 1.33	0.0109 ± 1.36	1.76 ± 0.90	2.37 ± 1.33	2.57 ± 1.36
Decrease detection threshold, ***w***_***det***_ by 100%	0.0136 ± 1.68	0.0133 ± 1.07	0.0128 ± 1.61	1.87 ± 1.08	2.38 ± 1.62	2.50 ± 1.68
Increase detection threshold, ***w***_***det***_ by 100%	0.0055 ± 1.33	0.0091 ± 1.54	0.010 ± 1.69	2.66 ± 1.29	2.25 ± 1.54	2.53 ± 1.69
Reduce minimum pest density, ***w***_***min***_ by 50%	0.0097 ± 1.27	0.013 ± 1.52	0.011 ± 1.73	2.61 ± 1.27	2.07 ± 1.52	2.42 ± 1.73
Increase minimum pest density, ***w***_***min***_ by 100%	0.0103 ± 1.93	0.0093 ± 1.48	0.0103 ± 1.70	2.53 ± 1.48	2.26 ± 1.70	2.81 ± 1.93
Increase budget, *B*, by 100%	0.0097 ± 1.95	0.0094 ± 1.94	0.0117 ± 2.37	3.60 ± 1.95	2.76 ± 1.94	5.04 ± 2.37
Higher fixed cost portion for management, *c*_*fix*_	0.0090 ± 1.09	0.0101 ± 1.64	0.0101 ± 1.73	1.92 ± 1.09	2.28 ± 1.64	2.43 ± 1.73
Lower fixed management cost, *c*_*fix*_ by 14% and increase variable cost, *c*_*var*_ by 25%	0.0091 ± 1.17	0.0098 ± 1.65	0.010 ± 1.78	2.08 ± 1.17	2.37 ± 1.65	2.78 ± 1.78
Decrease budget, *B*, by 17%	0.0089 ± 1.07	0.0105 ± 1.63	0.010 ± 1.68	1.87 ± 1.07	2.23 ± 1.63	2.31 ± 1.68
Decrease management efficiency, *e,* by 25%	0.0090 ± 1.18	0.010 ± 1.58	0.0104 ± 1.92	1.98 ± 1.18	2.21 ± 1.58	2.58 ± 1.92
Decrease budget, *B,* by 50%	0.0087 ± 0.88	0.011 ± 1.27	0.0104 ± 1.41	1.59 ± 0.88	1.78 ± 1.27	1.82 ± 1.41
Decrease budget, *B,* by 50% & increase detection threshold, ***w***_***det***_, by 100%	0.0128 ± 0.82	0.014 ± 1.33	0.0131 ± 1.22	1.44 ± 0.82	1.76 ± 1.33	1.69 ± 1.22

## Discussion

Our results have illustrated the advantages of multi-period planning in pest management programs. While the conventional, single-period reactive approach to manage the MPB infestation can substantially slow pest spread, it is far from strategically optimal long-term strategy. Short-sighted decisions only react to the most recent detections and cannot apply strategic forward-looking decisions to slow the long-distance spread over a longer term. Comparatively, even a mid-complexity model that factors in the simplest assessment of future spread without considering the impacts from future management actions helped to substantially reduce the infested area compared to the myopic model solutions. The mid-complexity model was also able to slow the exponential MPB spread to linear rate (Fig 3a). The relative simplicity of this approach makes it a good choice for practical use in situations where managers have limited access to expertise in optimization modelling.

The models differed substantially in how frequently they re-treated the same detected infested sites. Since the myopic model could not predict long-term spread trajectories, it could not anticipate which sites could be re-invaded in the future. Therefore, it could not make informed decisions on how frequently to treat a particular infested site. Comparatively, the full model was able to forecast the future outcomes of the treatment actions and applied strategic decisions to reduce the spread rate over a long term via frequent re-treatments of the sites that are most likely to contribute to long-distance spread in the future.

The myopic model tended to create the largest area infested with MPB. The exception was when the management budget level was set extremely large such that almost all high-risk sites could be treated. However, the idea of an unconstrained budget for a pest management program is unrealistic. In real-life conditions with a limited budget, the full model was superior. A critical advantage of the full multi-period formulation is that it can track pest detection lags by forecasting both the future outcomes of current infestations and the impacts of the management decisions on the population growth and future long-distance spread. The latter aspect enabled the early detection of ‘hub’ sites that would disproportionally contribute to the creation of remote infestation nuclei via long-distance spread and helped refocus tree removal efforts on these hubs at the earlier stages of infestation immediately after detection.

Our sensitivity analyses revealed that the relative ranking of model strategies was relatively insensitive to specific MPB invasion dynamics and detectability parameters, and the myopic model only performed similarly to the more complex models when the treatment budgets were set unrealistically large. More effective management than our baseline model scenario may lead to a slight overprediction of mid-complexity model performance compared to the myopic model. Slightly increasing the budget allocation for management could be a viable management strategy that would help account for more dispersers than the current budget level allows. Lower MPB infestation detectability than assumed in our baseline scenario may also imply that some sites should be re-treated more frequently than our baseline solutions predict for optimal performance. However, none of these changes could alter the qualitative performance improvement of our more complex models vs. the myopic model. Across the parameters tested, it is beneficial to stop treating core invasion sites in favour of stamping out long-distance invasion nuclei.

### Improving the management of MPB infestation

Our work emphasizes the benefits of strategic long-term planning of the MPB management efforts for slowing its spread across Alberta and delaying the incursion to Saskatchewan. Our results indicate that a spatial management strategy with long-term forecast horizons allows some forest losses in core areas with already established MPB populations but more effectively slows the eastward spread from newly infested patches near the leading edge of the infestation. This trade-off would also help reduce the overall pine mortality in Western Canada. Our results indicate that the strategic planning of MPB treatment efforts, if incorporated into management strategies, could have resulted in a total halt of eastward MPB spread during the time horizon covered by our model.

In recent years, eastward expansion of MPB has halted and even contracted in nearly all locations [54]. This is likely due in part to the impact of the SMAC program. Even if there are obvious limits on how much could be invested into the development of analytical expertise and computational capacity required to apply the full multi-period model, simpler projections of future spread rates without tracking feedback from future management efforts (as exemplified by our mid-complexity model scenarios) would already be more cost-effective than the short-sighted reaction to recent detections. However, we note that the mid-complexity approach sometimes struggled to make full use of the available treatment budget toward the end of planning horizon due to its poor capacity to detect short-term gains from future management decisions when not accounting for alterations in spread. This issue could be alleviated by extending the forecast of the spread only period a few years beyond the end of the planning horizon, *T*.

### Canadian pest management agencies and decision support tools

The existing guidance on MPB management stresses the importance of early, aggressive and sustained detection and tree removal efforts and instructs land management agencies to send clear and consistent messages stating that “If you’re not early, you’re too late” [26]. We reproduced this approach with our myopic model, which tended to manage all selected sites similarly according to the suggested early response strategy. Our results corroborate that a short-sighted prioritisation of newly invaded sites is likely to result in a substantial reduction of the area infested by MPB compared to no-treatment scenarios. However, we show that a more complex, strategic approach could work even better, given the differential impact of aggressive management of the sites, which may disproportionally contribute to the future long-distance spread.

Our results indicate that a more complex multi-period planning approach yields a more effective slow-the-spread strategy. In practical terms, The Province of Alberta generally followed two objectives in its MPB program ordered by priority: 1) Contain infestations and minimize spread of MPB north and south along the eastern slopes of the Rocky Mountains in Alberta; and 2) prevent the spread of MPB eastward into the boreal forest [55]. These priorities reflect that jack pine forests in eastern Alberta are not as sought after as lodgepole pine in western Alberta as a lumber commodity. The myopic or mid-complexity model could be sufficient if slowing the long-distance spread is not the prime management objective. We also note that the range of MPB has likely retracted in Alberta and has not spread as far east as was predicted by earlier modelling efforts [54]. Viewed in this context, our estimates of MPB’s spread potential based on historical epidemic spread rates may be somewhat conservative, with a larger predicted spread area than occurred. Nonetheless, we expect our MPB management models to provide similar relative benefits even in a restricted spread scenario. Future work could examine the longer-term management implications of MPB population crashes, the impacts of successive cold weather events and shifts to a lower-density endemic phase in this region.

Our optimisation models explored how managing a particular configuration of sites at the present time might impact spread rates in the future. We show that a simple anticipatory forecast, even without considering the full impact of present and future management decisions on spread, does a better job than a short-sighted reactive approach. This is because the anticipatory forecast informs which sites with early detections are more likely to be reinvaded and can then switch to a more expensive, aggressive response. This model demonstrates how limited resources could be redirected to managing sites that are likely to provide the largest contributions to future spread. For example, our mid-complexity approach can be considered as a lower benchmark when planning the allocation of MPB treatments, as it will require less expertise and is computationally simpler. In fact, any effort to forecast future pest spread based on current management efforts could have substantial benefits to the planning of future management actions.

Our most complex model is likely out of scope for use in on-the-ground management, which is why we explored how much benefit there was in modelling spread without incorporating the impact of future management on spread via our mid-complexity model. We note the lesser research effort may be required in using R packages such as *prioritizr* [56] relative to our more complex optimization-based formulation for this type of model. Future work could implement our prioritisation based on future spread in an R wrapper model for more direct uptake by managers.

### Potential model applications

Our modelling approach can be applied to other pest species and decision-making scenarios. Bark beetles are common invasive species worldwide and require the development of analogous management strategies across their invaded ranges [57]. North American pine species are planted around the world in commercial tree plantations, and so this work could help develop a management response should mountain pine beetle be introduced into Australia or New Zealand [58,59]. It could also be used in eastern North America should the range of MPB continue to expand. Beyond pests, our modelling approach could help inform ongoing efforts in long-term spatial conservation planning that account for the landscape configuration of threatened species who rely on connected, suitable habitat for dispersal, including in response to climate change [60]. Many existing planning approaches for integrating landscape configuration do so in a static way [56,61], rather than being able to account for the effects of spatial co-management in other sites on long term dispersal.

Our models and sensitivity analyses did not account for differences in MPB dispersal dynamics among pine species. For example, MPB populations were thought to behave roughly equivalently in jack pine and hybrid jack pine-lodgepole pine forests as they do in pure lodgepole pine [10]. Only recently has MPB’s eruptive potential in jack pine been shown to be lower [28]. However, we do not anticipate that accounting for pine species-specific MPB behaviour would qualitatively change the best model type for management, as MPB growth dynamics are similar among tree species. More importantly, managers do not vary their tree removal strategy according to which pine species is being attacked by the pest. While the region invaded by MPB distribution in our study area was not dominated by jack pine, accounting for pine species differences would be important extension to our work if the model is to be applied in eastern Canada, given its broader taxonomic diversity of pine species.

We have assumed consistent climate dynamics over the region and time period simulated across the model scenarios. This simplification was less problematic over the decade-long time horizon. However, the transition of MPB outbreaks to their epidemic phases can be influenced by environmental conditions. A warming climate is expected to promote synchronous adult emergence and further increase the likelihood of successful mass attack [1]. Warmer winters may also reduce MPB’s overwintering mortality, which partially explains the northward and eastward shifts in MPB’s distribution range [20,62,63]*.* Warming may become detrimental to MPB beyond a threshold, as it can accelerate development to less cold-hardy adult stages, increasing mortality risk [64]. Beyond temperature, drought stress could potentially reduce host tree defensive capacities, making it easier for beetles to overcome Allee effects [64]. Notably, our model could be easily extended to include site specific climatic predictors of the population growth and minimum density levels on propagule arrival. This would be an important focus for future research.

Our model also assumed dispersal as a climatically independent process. Our choice of isotropic dispersal kernel was meant to depict a longer term, large-scale average dispersal behaviour. In reality, individual long-distance MPB dispersal events could occur during short periods of warm, fair weather, and spread the beetles in the dominant wind direction at that time [1]. This additional complexity is computationally complex in a multiperiod problem formulation, but has been explored in recent dispersal models [41], and could be incorporated in future work.

### Putting a cost on managing reactively

We argue that the benefits of strategically managing an expanding pest population become especially obvious over the long term. A lack of long-term forecasting of MPB spread rates and the future impacts of the management decisions may obscure the long-term benefits of the treatment efforts aimed to slow MPB spread. Our results indicate that managers might not always detect the immediate benefits of changing their management strategy. However, the anticipatory nature of the strategic planning approach would yield a better reduction of the spread rate in the long term. Given the challenge of forecasting rare, long-distance spread events, future studies should examine the sensitivity of our findings to the uncertainty about long-distance spread forecasts. The notion that spending resources to manage an invasion early would pay dividends over the long term is well understood, but it is challenging to quantify those potential dividends in a decision-making context and communicate their magnitude to practitioners [65]. In fact, this aspect has been posited as a major reason for a global lack of proactive biosecurity expenditures [66]. Our approach helps quantify the explicit cost of the short-sighted reactive management versus the strategic management decisions and thus helps incentivise more proactive approaches to manage biological invasions.

## Supporting information

S1 Appendix Additional spread maps. (DOCX)

S2 Appendix A linearized formulation of the full MPB optimisation problem. (DOCX)

S3 Appendix Results from sensitivity analyses. (DOCX)
