# Supplementary material for: The value of looking ahead: Comparing conventional and strategic Mountain Pine Beetle (Dendroctonus ponderosae) management policies in North America
Source: PLoS One. 2026 Jun 24;21(6):e0344860. doi: 10.1371/journal.pone.0344860 (PMC13293515; doi:10.1371/journal.pone.0344860)
Supplement: S1 Appendix — (DOCX) [file pone.0344860.s001.docx]

**Appendix 1. Additional spread maps**

**Figure S1. Example of the binary spread pattern (in black) from site *i*=183 (in yellow) in period *t*=1.**The rest of the simulation region in shown in a grid, with the Saskatchewan border shown as a red line. Basemap containsinformation from Statistics Canada, licensed under Open Government Licence – Canada, from R package *canadamaps*v2.0.0 (Cayen 2024).
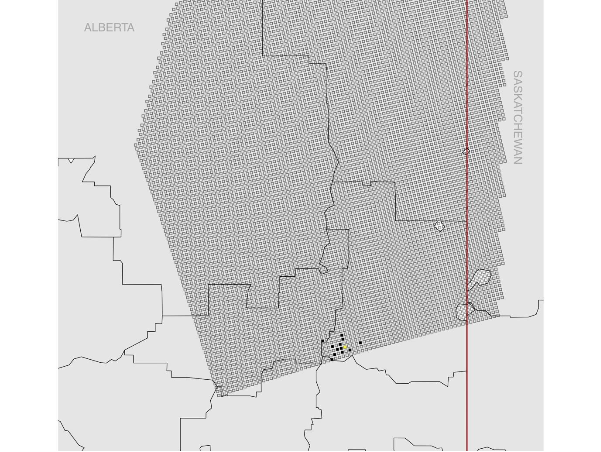


**Figure S2. Maps of MPB spread over the planning horizon *T***: **a)** myopic model; **b)** mid-complexity model; **c)**full multi-period model. The pest population density status: undetectable (*w_i_<w_min_*), detectable and manageable (*w_spr_>w_i_>w_min_*), capable of long-distance spread (*w_max_>w_i_>w_spr_*), and collapsed (*w_i_>w_max_*). The Alberta-Saskatchewan border is shown as a vertical line. Basemap contains information from Statistics Canada, licensed under Open Government Licence – Canada, from R package *canadamaps*v2.0.0 (Cayen 2024).
