# Supplementary material for: The value of looking ahead: Comparing conventional and strategic Mountain Pine Beetle (Dendroctonus ponderosae) management policies in North America
Source: PLoS One. 2026 Jun 24;21(6):e0344860. doi: 10.1371/journal.pone.0344860 (PMC13293515; doi:10.1371/journal.pone.0344860)
Supplement: S2 Appendix — (DOCX) [file pone.0344860.s002.docx]

**Appendix 2. A linearized formulation of the full MPB optimisation problem.**

**Sets:**

*I* – sites *i, j, k* with susceptible host trees suitable to support the pest population, *i, j, k* ∈ *I*;

*T* – planning periods *t, t* ∈ *T*.

**Decision variables:**

*w_it_, w_ij_* ∈ [0;1] *–* population density in site *i* in period *t*, adjusted by *w_i_* _max_;

*w’_it_, w’_ij_* ∈ [0; *y_i_*] *–* population density in site *i* in period *t* after treatment and population growth, adjusted by *w_i_* _max_;

*w’’_it_, w’’_ij_* ∈ [0;*U*] *–* population density in site *i* in period *t*, adjusted by spread from / to other sites;

*u_it_*, *u_it_* ∈ {0,1}– binary indicator of that site *i* is infested in period *t* (*u_it_*=1 for *w_it_*>*w*_min_ and *u_it_*=0 otherwise);

*v_it_*, *v_it_* ∈ {0,1} *–* binary indicator of that the infestation is detectable in site *i* in period *t (v_it_* = 1 and *v_it_* = 0 otherwise);

*q_it_*, *q_it_* ∈ {0,1} *–* binary indicator that site *i* is treated in period *t* (*q_it_ =* 1 and *q_it_ = 0* otherwise);

*λ_it_*, *λ_it_* ∈ {0,1} – binary indicator that the population density in site *i* in period *t* is above the dispersal threshold, *w_spr_* , and below the threshold for population collapse, *w_max_* (i.e., *λ_it_* = 1). The population in site *i* with the density below the dispersal threshold cannot spread propagules to other sites (e.g., *λ_it_* = 0);

*d_ij_*, *d_ij_* ∈ {0,1} – binary indicator that the population density in site *i* in period *t* is above the threshold for population collapse, *w_max_* (i.e., *h_it_* = 1). The population in site *i* with the density above this threshold cannot disperse propagules to other sites (e.g., *λ_it_* = 1)

*n_it_*, *n_it_* ∈ {0,1} *–* binary indicator of that the population density in site *i* in period *t* is below the carrying capacity *w_i_* _max_ (*n_it_* = 1 and *n_it_* = 0 otherwise) – needed to calculate the *w’_it_* values;

*m_it_*, *m_it_* ∈ {0,1} *–* binary indicator of that the population density in site *i* in period *t* is below the carrying capacity *w_i_* _max_ (*m_it_* = 1 and *m_it_* = 0 otherwise) – needed to calculate the *w_i t+_*_1_ values;

*g_it_*, *g_it_* ∈ [0;1] *–* linearizing the product of a binary variable *q_it_* and a non-negative variable *w_it_*;

*l_it_*, *l_it_* ∈ {0,1} *–* linearizing the product of binary variables *n_it_* and *q_it_*;

*s_it_*, *s_it_* ∈ [0;1] *–* linearizing the product of a binary variable *m_it_* and a non-negative variable *w’’_it_*;

*x_it_*, *x_it_* ∈ [0;1] *–* linearizing the product of a binary variable *n_it_* and a non-negative variable *w_it_*.

*z_it_*, *z_it_* ∈ [0;1] *–* linearizing the product of a binary variable *h_it_* and a non-negative variable *w_it_*;

*p_it_*, *p_it_* > 0 – linearizing the product of the survey selection binary variable *q_it_*, and the population density variable *w’_it_*. We assume that the treatment cost of the densities above the detection density threshold *w_i_* _det_ required the removal of completely infested tress and so the population density within the interval [*w_i_* _det_; *w*_treat_] was linearly proportional to the number of completely infested (and so detectable) host trees;

*z_it_*, *z_it_* ∈ [0;1] *–*linearizing the product of binary variables *h_it_* and a non-negative variable *w_it_*;

*δ_it_*, *δ_it_* ∈ {0,1} *–*linearizing the product of binary variables *λ_it_* and *d_it_*;

*φ_it_*, *φ_it_* ∈ {0,1}*–* linearizing the product of binary variables *λ_it_* and *v_it_*;

$\beta$*_it_*, $\beta$*_it_* ∈ {0,1}*–* linearizing the product of binary variables *φ_it_* and *d_it_*;

$\gamma$*_it_*, $\gamma$*_it_* ∈ [0;1]*–* linearizing the product of a binary variable *u_it_* and the non-negative variable *w’_it_*;

**Parameters:**

*B, B* > 0 – the treatment budget limit for management area *I* in period *t*;

*f* , *f >0* – scaling factor;

*D_ijt_, D_ijt_* ∈ {0,1} – binary indicator that site *i* can spread propagules to other sites *j* if it has the pest population density *w’_it_* above the threshold *w*_spr_ in period *t*;

*M, M* > 0 – big-M value;

*e* , 0< *e* ≤ 1 ‑ proportion of a 5×5-km site treated in period *t* (a metric of treatment efficiency);

*c_fix_* ,*c_fix_* > 0 ‑ fixed portion of the treatment cost (independent of the MPB density in site *i*);

*c_var_* ,*c_var_* > 0 ‑ variable portion of the treatment cost (proportional to the MPB density in site *i*);

*y_i_, y_i_ >* 1 – annual population growth rate in site *i; y_i_* > 1;

*w*_spr_, 0<*w*_spr_ ≤ 1 – minimum population density in site *i* when propagules can spread to other sites *j*;

*w*_max_, 0< *w*_max_ ≤ 1 – maximum population density before it stops producing propagules (relative to carrying capacity of 1).

*w_i_* _det_ *, w_i_* _det_ ∈ ]0;1] *–* minimum population density at which the infestation in site *i* can be detected;

*w*_min_; *w* _min_ ∈ ]0;1] – minimum population density at a newly infested site;

*w*_min_ > 0; *w*_min_ < *w_i_* _det_; *w_i_* _det_ < *w*_spr_ < *w*_max_; *w*_max_ ≤ 1.

**Equations:**

$\min\sum_{i=1}^{I} \sum_{t=1}^{T} v_{it}+fu_{it}$ (minimize the detected infested area over *T* periods, plus a small penalty for undetected infested sites) [1]

*s.t.*:

*Defining the infested sites:*

*u_it_ ≥ (w_it_ –* *w*_min_) ∀ *i* ∈ *I* , *t* ∈*T (*site *i* is “infested” in period *t* if *w_it_*≥*w*_min_) [2]

*u_it_ ≤ w_it_/w*_min_ ∀ *i* ∈ *I* , *t* ∈*T* (site *i* is not considered “infested” in period *t* if *w_it_ ≤ w*_min_) [3]

*Detectability constraints:*

*q_it_* ≤ *v_it_* ∀ *i* ∈ *I* , *t* ∈*T* (only the detectable infestations can be treated) [4]

*v_it_* ≥ (*w_it_* – *w*_det_) ∀ *i* ∈ *I* , *t* ∈*T* (detectability indicator: *v_it_* = 1 when *w_it_* > *w*_det_) [5]

*v_it_* ≤ *w_it_*/*w*_det_  ∀ *i* ∈ *I* , *t* ∈*T* (detectability indicator: *v_it_* = 0 when *w_it_* ≤ *w*_det_) [6]

*Budget constraint*

$\sum_{i=1}^{I} q_{it}{(c_{fix}+c_{var}w'}_{it}) \leq B$∀ *t* ∈*T or, assuming*$p_{it}={w'}_{it}q_{it}$ [7]

$\sum_{i=1}^{I} q_{it}{c_{fix}+c_{var}p}_{it} \leq B$∀ *t* ∈*T* [8]

*Initial population density:*

*w_i_*_1_ = *w_i_* _init_ ∀ *i* ∈ *I* (set the population density in site *i* in period *t* = 1 to *w_i_* _init_) [9]

*On-site population growth in site i in period (limited by carrying capacity w_i_* _max_*)*:

*w'_it_* = *y_i_n_it_w_it_*(1 – *q_it_e*) + (1 – *n_it_*)*w_i_* _max_ ∀ *i* ∈ *I* , *t*∈ *T* [10]

*w’_it_* = *y_i_n_it_w_it_* – *y_i_n_it_w_it_q_it_e* + (1 – *n_it_*)*w*_max_ ∀ *i* ∈ *I* , *t*∈ *T*

or, assuming *z_it_ = n_it_w_it_q_it_* and *x_it_* = *n_it_w_it_*:

*w’_it_* = *y_i_x_it_* – *y_i_z_it_e* + (1 – *n_it_*)*w*_max_ ∀ *i* ∈ *I* , *t*∈ *T*  (population growth in site *i* in period *t*) [11]

*n_it_* ≤ 1 + (*w*_max_ – *y_i_w_it_*(1– *q_it_e*))/M ∀ *i* ∈ *I* , *t* ∈*T* [12]

*n_it_* ≤ 1 + (*w*_max_ – *y_i_(w_it_* - *w_it_q_it_e*))/M ∀ *i* ∈ *I* , *t* ∈*T* or, assuming *g_it_* = *w_it_q_it_*: [13]
*n_it_* ≤ 1 + (*w*_max_ – *y_i_(w_it_* - *g_it_e*)/M ∀ *i* ∈ *I* , *t* ∈*T* (*n_it_*=0 when *w_it_* > *w_i_* max) [14]

*n_it_* ≥ 1 – (*y_i_w_it_*(1 – *q_it_e*))/*w_i_* _max_ ∀ *i* ∈ *I* , *t* ∈*T*  [15]

*n_it_* ≥ 1 – *y_i_(w_it_* – *g_it_e*))/*w_i_* _max_ ∀ *i* ∈ *I* , *t* ∈*T* (*n_it_*=1 when *w_it_* ≤ *w_i_* max) [16]

*Dispersal to uninfested (below w_min_) sites from sites above threshold w_spr_ (limited by collapse threshold w_i_* _max_ *)*:

${w''}_{it}=\left( 1-v_{it} \right)w_{min}\max_{j} {[D}_{ji}l_{it}d_{jt}] + {w^{'}}_{it}u_{it} \forall i\in I, t\in T$ or: [17]

${w''}_{it}=w_{min}D_{ji}l_{it}d_{jt}-w_{min}\max_{j} {[D}_{ji}l_{it}d_{jt}v_{it}] + {w^{'}}_{it}u_{it} \forall i\in I, t\in T$ [18]

or, assuming $d_{it}=l_{it}d_{jt}$, ${\beta_{it}=l}_{it}d_{jt}v_{it}$, and $\gamma_{it}{=w^{'}}_{it}u_{it}$

${w''}_{it}=w_{min}D_{ji}d_{it}-w_{min}\max_{j} {[D}_{ji}\beta_{it}]+ \gamma_{it}\forall i\in I, t\in T$ [19]

*Dispersal threshold (dispersal only above spread threshold and below upper population collapse threshold):*

*d_it_* ≥ *w’_it_ – w_i max_* ∀ *i* ∈ *I* , *t* ∈*T* [20]

*d_it_* ≤ *w’_it_*/ *w_i max_* ∀ *i* ∈ *I* , *t* ∈*T*  [21]

*λ_it_* ≥ *w’_it_* – $w_{{spr}_{i}}$ – *d_it_* ∀ *i* ∈ *I* , *t* ∈*T*  [22]

*λ_it_* ≤ *w’_it_*/$w_{{spr}_{i}}$ ∀ *i* ∈ *I* , *t* ∈*T* [23]

*λ_it_*≤ *1 –* *d_it_* [24]

*Limit the population density at the next timestep by the carrying capacity 1*:

*w_i t_*_+1 =_ *m_it_w’’_it_* + (1 – *m_it_*) ∀ *i* ∈ *I*, *t* = 1,...,*T –* 1 or, assuming *s_it=_ m_it_w’’_it_* [25]

*w_i t_*_+1 =_ *s_it_* + (1 – *m_it_*)*w_i_* _max_ ∀ *i* ∈ *I*, *t* = 1,...,*T –* 1 (population density in site *i* in period *t* + 1) [26]

*m_it_* ≤ 1 + (*1* – *w’’_it_*)/ *M* ∀ *i* ∈ *I* , *t* ∈*T*  [27]

*m_it_* ≥ 1 – *w’’_it_* ∀ *i* ∈ *I* , *t* ∈*T*  [28]

*Linearizing the products of decision variables*:

*g_it_* ≤ *q_it_* ∀ *i* ∈ *I*, *t* ∈*T*  (the product of *w_it_* and *q_it_*) [29]

*g_it_* ≤ *w_it_* ∀ *i* ∈ *I*, *t* ∈*T*  (the product of *w_it_* and *q_it_*) [30]

*g_it_* ≥ *w_it_* – (1 – *q_it_*) ∀ *i* ∈ *I*, *t* ∈*T* (the product of *w_it_* and *q_it_*) [31]

*x_it_* ≤ *n_it_* ∀ *i* ∈ *I*, *t* ∈*T*  (the product of *w_it_* and *n_it_*) [32]

*x_it_* ≤ *w_it_* ∀ *i* ∈ *I*, *t* ∈*T*  (the product of *w_it_* and *n_it_*) [33]

*x_it_* ≥ *w_it_* – (1 – *n_it_*) ∀ *i* ∈ *I*, *t* ∈*T* (the product of *w_it_* and *n_it_*) [34]

*l_it_* ≤ *n_it_* ∀ *i* ∈ *I*, *t* ∈*T*  (the product of *n_it_* and *q_it_*) [35]

*l_it_* ≤ *q_it_* ∀ *i* ∈ *I*, *t* ∈*T*  (the product of *n_it_* and *q_it_*) [36]

*l_it_* ≥ *n_it_* + *q_it_* – 1 ∀ *i* ∈ *I*, *t* ∈*T*  (the product of *n_it_* and *q_it_*) [37]

*z_it_* ≤ *l_it_* ∀ *i* ∈ *I*, *t* ∈*T*  (the product of *w_it_* and *l_it_*) [38]

*z_it_* ≤ *w_it_* ∀ *i* ∈ *I*, *t* ∈*T*  (the product of *w_it_* and *l_it_*) [39]

*z_it_* ≥ *w_it_* – (1 – *l_it_*) ∀ *i* ∈ *I*, *t* ∈*T* (the product of *w_it_* and *l_it_*) [40]

*p_it_* ≤ *q_it_* ∀ *i* ∈ *I*, *t* ∈*T*  (the product of *w’_it_* and *q_it_*) [41]

*p_it_* ≤ *w’_it_* ∀ *i* ∈ *I*, *t* ∈*T*  (the product of *w’_it_* and *q_it_*) [42]

*p_it_* ≥ *w’_it_* – (1 – *q_it_*) ∀ *i* ∈ *I*, *t* ∈*T* (the product of *w’_it_* and *q_it_*) [43]

*s_it_* ≤ *Um_it_* ∀ *i* ∈ *I*, *t* ∈*T*  (the product of *w’’_it_* and *m_it_*) [44]

*s_it_* ≤ *w’’_it_* ∀ *i* ∈ *I*, *t* ∈*T*  (the product of *w’’_it_* and *m_it_*) [45]

*s_it_* ≥ *w’’_it_* – *U*(1 – *m_it_*) ∀ *i* ∈ *I*, *t* ∈*T* (the product of *w’’_it_* and *m_it_*) [46]

*δ_it_* ≤ *λ_it_* ∀ *i* ∈ *I*, *t* ∈*T*  (the product of *λ_it_* and *d_it_*) [47]

*δ_it_* ≤ *d_it_* ∀ *i* ∈ *I*, *t* ∈*T*  (the product of *λ_it_* and *d_it_*) [48]

*δ_it_* ≥ *λ_it_* + *d_it_* – 1 ∀ *i* ∈ *I*, *t* ∈*T*  (the product of *λ_it_* and *d_it_*) [49]

*φ_it_* ≤ *λ_it_* ∀ *i* ∈ *I*, *t* ∈*T*  (the product of *λ_it_* and *v_it_*) [50]

*φ_it_* ≤ *v_it_* ∀ *i* ∈ *I*, *t* ∈*T*  (the product of *λ_it_* and *v_it_*) [51]

*φ_it_* ≥ *λ_it_* + *v_it_* – 1 ∀ *i* ∈ *I*, *t* ∈*T* (the product of *λ_it_* and *v_it_*) [52]

$\beta$ *_it_* ≤ *φ_it_* ∀ *i* ∈ *I*, *t* ∈*T*  (the product of *φ_it_* and *d_it_*) [53]

$\beta$*_it_* ≤ *d_it_* ∀ *i* ∈ *I*, *t* ∈*T*  (the product of *φ_it_* and *d_it_*) [54]

$\beta$*_it_* ≥ *φ_it_* + *d_it_* –1 ∀ *i* ∈ *I*, *t* ∈*T* (the product of *φ_it_* and *d_it_*) [55]

$\gamma_{it}$≤ *w’_it_* ∀ *i* ∈ *I*, *t* ∈*T*  (the product of *w’_it_* and *u_it_*) [56]

$\gamma_{it}$≤ *u_it_* ∀ *i* ∈ *I*, *t* ∈*T*  (the product of *w’_it_* and *u_it_*) [57]

$\gamma_{it}$≥ *w’_it_* – (1 – *u_it_*) ∀ *i* ∈ *I*, *t* ∈*T*  (the product of *w’_it_* and *u_it_*) [58]

**Table S1. Model sets, decision variables and parameters.**

| 1. **Sets** | | |
| --- | --- | --- |
| **Symbol** | **Range** | **Description** |
| *I* | *i=*{*0,…,N*} | Sites (*i,j*) with susceptible host trees suitable to support the pest population |
| *T* | *t=*{*0,…,L*} | planning periods *t* |
| 1. **Decision variables** | | |
| **Symbol** | **Range** | **Description** |
| *w_it_* | *0≤w_it_≤1* | Relative value depicting the population density in site *i* in period *t* relative to the carrying capacity |
| *w’_it_,* | 0≤*w’_it_,≤y_i_* | Relative value depicting the population density in site *i* in period *t* relative to the carrying capacity after treatment and accounting for annual population growth |
| *w’’_it_* | 0≤ *w’’_it_* ≤M | Relative value depicting the population density in site *i* in period *t* relative to the carrying capacity, adjusted by spread of propagules from other sites to *i*; |
| *u_it_* | *u_it_* ={0,1} | Binary indicator of site *i* being infested in period *t* (*u_it_*=1 for *w_it_*>*w*_min_ and *u_it_*=0 otherwise) |
| *v_it_* | *v_it_* ={0,1} | Binary indicator of the infestation being detectable in site *i* in period *t (v_it_* = 1 and *v_it_* = 0 otherwise) |
| *q_it_* | *q_it_* ={0,1} | Binary indicator of site *i* being treated in period *t* (*q_it_ =* 1 and *q_it_ = 0* otherwise) |
| *λ_it_* | *λ_it_* ={0,1} | Binary indicator of the pest population density in site *i* in period *t* being both above the dispersal threshold, *w_spr_* , and below the threshold for population collapse, *w_max_* (i.e., *λ_it_* = 1). The population in site *i* with the density level below the dispersal threshold cannot spread propagules to other sites *j* (e.g., *λ_it_* = 0) |
| *d_ij_* | *d_ij_* ={0,1} | Binary indicator of the pest population density in site *i* in period *t* being above the threshold for population collapse, *w_max_* (i.e., *d_it_* = 1). The population in site *i* with the density above this threshold cannot disperse propagules to other sites (e.g., *λ_it_* = 1) |
| *n_it_* | *n_it_* ={0,1} | Binary indicator of the population density in site *i* in period *t* being below the carrying capacity *w_i_* _max_ (*n_it_* = 1 and *n_it_* = 0 otherwise) – needed to calculate *w’_it_* |
| *m_it_* | *m_it_* ={0,1} | Binary indicator of the population density in site *i* in period *t* being below the carrying capacity *w_i_* _max_ (*m_it_* = 1 and *m_it_* = 0 otherwise) – needed to calculate *w_i t+1_* |
| 1. **Parameters** | | |
| **Symbol** | **Range** | **Description** |
| *B* | B>0 | An upper bound budget limit to treat the management area *I* in period *t* (where B=1 is the cost of treating 1 cell at carrying capacity). B=150 in the baseline scenario. |
| *f* | *f*>0 | Scaling factor for prioritizing the impact of non-detected sites in the objective function value. *f=*0.0001 in all scenarios. |
| *h_i_* | *0<h*≤1 | Relative value depicting the host density at site *I* |
| *r_ij_* | *r>0* | Euclidean distance between sites *i* and *j* (in units of 5 km wide cells) |
| *D_ijt_* | *D_ijt_ ={0,1}* | Binary indicator that defines that propagules can spread from site *i* elsewhere in period *t* if the population density in *i*, *w’_it_,* is above the spread threshold, *w*_spr_. |
| *M* | *M>0* | Large positive value (Big-M value in the indicator constraints) in units of relative population density. |
| *N* | *N*=7925 | Total number of sites *i* |
| *L* | L=*11* | The number of periods *t* (*yr*) |
| *e* | 0≤ *e*≤1 | The proportion of site *i* that is treated in period *t* (a surrogate of the treatment efficiency in *i*) |
| *c_fix_* | *c_fix_>0* | A fixed portion of the management costs that is independent of the density of the MPB population in site *I* |
| *c_var_* | *c_var_* > 0 | A variable component of the management cost that is proportional to the density of MPB population in site *i* in period *t* |
| *y_i_* | *y_i_ >* 1 | The annual population growth rate in site *i (yr^-1^)*. *y=*1.45±30% in all model scenarios. |
| *w*_i spr_ | 0< *w*_i spr_≤1 | The minimum population density level when propagules can disperse from site *i* to other sites. *w_ispr_=*0.022±30% in all model scenarios. |
| *w*_max_ | 0< *w*_i max_≤1 | The density level that population can reach before it stops sending out propagules (relative to the carrying capacity of 1). *w_max_=*1 in all model scenarios. |
| *w*_det_ | 0< *w*_i det_≤1 | The minimum population density level when the infestation in site *i* can be detected (relative to the carrying capacity of 1). *w*_det_=0.008 in the baseline scenario. |
| *w*_min_ | 0< *w*_min_≤1 | The population density level for newly infested sites (relative to the carrying capacity of 1). *w*_min_=0.004 in the baseline scenario. |
