# Supplementary material for: The value of looking ahead: Comparing conventional and strategic Mountain Pine Beetle (Dendroctonus ponderosae) management policies in North America
Source: PLoS One. 2026 Jun 24;21(6):e0344860. doi: 10.1371/journal.pone.0344860 (PMC13293515; doi:10.1371/journal.pone.0344860)
Supplement: S3 Appendix — (DOCX) [file pone.0344860.s003.docx]

**Appendix 3**. **Results from sensitivity analyses.**

1. Increase management efficiency by 30%

**Fig S3.** The frequency of treatment for mountain pine beetle infestations (number of times each site was managed) during the entire planning horizon for the single period (myopic) spread-only (mid-complexity) and multiperiod (full) models in the baseline scenario. The Saskatchewan border is shown by a dark red vertical line. Each square represents a 5×5 km cell in the model.

**Fig S4.** The difference in relative infestation time of forests by mountain pine beetle between the single period (myopic) and multiperiod (full) model solutions. See text for description of model solutions The initial area invaded by the insect at *t=1* is shown in grey. Each square represents a 5×5 km cell in the model. Cells that were never infested in one model type were infested in the other model type. Basemap contains information from Statistics Canada, licensed under Open Government Licence – Canada, from R package *canadamaps* v2.0.0 (Cayen 2024).

1. Decrease detection threshold by 100%

**Fig S5.** The frequency of treatment for mountain pine beetle infestations (number of times each site was managed) during the entire planning horizon for the single period (myopic) spread-only (mid-complexity) and multiperiod (full) models in the baseline scenario. The Saskatchewan border is shown by a dark red vertical line. Each square represents a 5×5 km cell in the model.

**Figure S6.** The difference in relative infestation time of forests by mountain pine beetle between the single period (myopic) and multiperiod (full) model solutions. See text for description of model solutions The initial area invaded by the insect at *t=1* is shown in grey. Each square represents a 5×5 km cell in the model. Cells that were never infested in one model type were infested in the other model type. Basemap contains information from Statistics Canada, licensed under Open Government Licence – Canada, from R package *canadamaps* v2.0.0 (Cayen 2024).

1. Increase detection threshold by 100%

**Fig S7.** The frequency of treatment for mountain pine beetle infestations (number of times each site was managed) during the entire planning horizon for the single period (myopic) spread-only (mid-complexity) and multiperiod (full) models in the baseline scenario. The Saskatchewan border is shown by a dark red vertical line. Each square represents a 5×5 km cell in the model.

**Figure S8.** The difference in relative infestation time of forests by mountain pine beetle between the single period (myopic) and multiperiod (full) model solutions. See text for description of model solutions The initial area invaded by the insect at *t=1* is shown in grey. Each square represents a 5×5 km cell in the model. Cells that were never infested in one model type were infested in the other model type. Basemap contains information from Statistics Canada, licensed under Open Government Licence – Canada, from R package *canadamaps* v2.0.0 (Cayen 2024).

1. Reduce minimum pest density by 100%

**Fig S9.** The frequency of treatment for mountain pine beetle infestations (number of times each site was managed) during the entire planning horizon for the single period (myopic) spread-only (mid-complexity) and multiperiod (full) models in the baseline scenario. The Saskatchewan border is shown by a dark red vertical line. Each square represents a 5×5 km cell in the model.

**Figure S10.** The difference in relative infestation time of forests by mountain pine beetle between the single period (myopic) and multiperiod (full) model solutions. See text for description of model solutions The initial area invaded by the insect at *t=1* is shown in grey. Each square represents a 5×5 km cell in the model. Cells that were never infested in one model type were infested in the other model type. Basemap contains information from Statistics Canada, licensed under Open Government Licence – Canada, from R package *canadamaps* v2.0.0 (Cayen 2024).

1. Increase minimum pest density by 100%

**Fig S11.** The frequency of treatment for mountain pine beetle infestations (number of times each site was managed) during the entire planning horizon for the single period (myopic) spread-only (mid-complexity) and multiperiod (full) models in the baseline scenario. The Saskatchewan border is shown by a dark red vertical line. Each square represents a 5×5 km cell in the model.

**Figure S12.** The difference in relative infestation time of forests by mountain pine beetle between the single period (myopic) and multiperiod (full) model solutions. See text for description of model solutions The initial area invaded by the insect at *t=1* is shown in grey. Each square represents a 5×5 km cell in the model. Cells that were never infested in one model type were infested in the other model type. Basemap contains information from Statistics Canada, licensed under Open Government Licence – Canada, from R package *canadamaps* v2.0.0 (Cayen 2024).

1. Increase budget by 100%

**Fig S13.** The frequency of treatment for mountain pine beetle infestations (number of times each site was managed) during the entire planning horizon for the single period (myopic) spread-only (mid-complexity) and multiperiod (full) models in the baseline scenario. The Saskatchewan border is shown by a dark red vertical line. Each square represents a 5×5 km cell in the model.

**Figure S14.** The difference in relative infestation time of forests by mountain pine beetle between the single period (myopic) and multiperiod (full) model solutions. See text for description of model solutions The initial area invaded by the insect at *t=1* is shown in grey. Each square represents a 5×5 km cell in the model. Cells that were never infested in one model type were infested in the other model type. Basemap contains information from Statistics Canada, licensed under Open Government Licence – Canada, from R package *canadamaps* v2.0.0 (Cayen 2024).

1. Higher fixed cost portion for management

**Fig S15.** The frequency of treatment for mountain pine beetle infestations (number of times each site was managed) during the entire planning horizon for the single period (myopic) spread-only (mid-complexity) and multiperiod (full) models in the baseline scenario. The Saskatchewan border is shown by a dark red vertical line. Each square represents a 5×5 km cell in the model.

**Figure 16.** The difference in relative infestation time of forests by mountain pine beetle between the single period (myopic) and multiperiod (full) model solutions. See text for description of model solutions The initial area invaded by the insect at *t=1* is shown in grey. Each square represents a 5×5 km cell in the model. Cells that were never infested in one model type were infested in the other model type. Basemap contains information from Statistics Canada, licensed under Open Government Licence – Canada, from R package *canadamaps* v2.0.0 (Cayen 2024).

1. Lower fixed management cost by 14% and increase variable management cost by 25%

**Fig S17.** The frequency of treatment for mountain pine beetle infestations (number of times each site was managed) during the entire planning horizon for the single period (myopic) spread-only (mid-complexity) and multiperiod (full) models in the baseline scenario. The Saskatchewan border is shown by a dark red vertical line. Each square represents a 5×5 km cell in the model.

**Figure S18.** The difference in relative infestation time of forests by mountain pine beetle between the single period (myopic) and multiperiod (full) model solutions. See text for description of model solutions The initial area invaded by the insect at *t=1* is shown in grey. Each square represents a 5×5 km cell in the model. Cells that were never infested in one model type were infested in the other model type. Basemap contains information from Statistics Canada, licensed under Open Government Licence – Canada, from R package *canadamaps* v2.0.0 (Cayen 2024).

1. Decrease budget by 17%

**Fig S19.** The frequency of treatment for mountain pine beetle infestations (number of times each site was managed) during the entire planning horizon for the single period (myopic) spread-only (mid-complexity) and multiperiod (full) models in the baseline scenario. The Saskatchewan border is shown by a dark red vertical line. Each square represents a 5×5 km cell in the model.

**Figure S20.** The difference in relative infestation time of forests by mountain pine beetle between the single period (myopic) and multiperiod (full) model solutions. See text for description of model solutions The initial area invaded by the insect at *t=1* is shown in grey. Each square represents a 5×5 km cell in the model. Cells that were never infested in one model type were infested in the other model type. Basemap contains information from Statistics Canada, licensed under Open Government Licence – Canada, from R package *canadamaps* v2.0.0 (Cayen 2024).

1. Decrease efficiency by 25%

**Fig S21.** The frequency of treatment for mountain pine beetle infestations (number of times each site was managed) during the entire planning horizon for the single period (myopic) spread-only (mid-complexity) and multiperiod (full) models in the baseline scenario. The Saskatchewan border is shown by a dark red vertical line. Each square represents a 5×5 km cell in the model.

**Figure S22.** The difference in relative infestation time of forests by mountain pine beetle between the single period (myopic) and multiperiod (full) model solutions. See text for description of model solutions The initial area invaded by the insect at *t=1* is shown in grey. Each square represents a 5×5 km cell in the model. Cells that were never infested in one model type were infested in the other model type. Basemap contains information from Statistics Canada, licensed under Open Government Licence – Canada, from R package *canadamaps* v2.0.0 (Cayen 2024).

1. Decrease budget by 50%

**Fig S23.** The frequency of treatment for mountain pine beetle infestations (number of times each site was managed) during the entire planning horizon for the single period (myopic) spread-only (mid-complexity) and multiperiod (full) models in the baseline scenario. The Saskatchewan border is shown by a dark red vertical line. Each square represents a 5×5 km cell in the model.

**Figure S24.** The difference in relative infestation time of forests by mountain pine beetle between the single period (myopic) and multiperiod (full) model solutions. See text for description of model solutions The initial area invaded by the insect at *t=1* is shown in grey. Each square represents a 5×5 km cell in the model. Cells that were never infested in one model type were infested in the other model type. Basemap contains information from Statistics Canada, licensed under Open Government Licence – Canada, from R package *canadamaps* v2.0.0 (Cayen 2024).

1. Decrease budget by 50% & increase detection threshold by 100%

**Fig S25.** The frequency of treatment for mountain pine beetle infestations (number of times each site was managed) during the entire planning horizon for the single period (myopic) spread-only (mid-complexity) and multiperiod (full) models in the baseline scenario. The Saskatchewan border is shown by a dark red vertical line. Each square represents a 5×5 km cell in the model.

**Figure S26.** The difference in relative infestation time of forests by mountain pine beetle between the single period (myopic) and multiperiod (full) model solutions. See text for description of model solutions The initial area invaded by the insect at *t=1* is shown in grey. Each square represents a 5×5 km cell in the model. Cells that were never infested in one model type were infested in the other model type. Basemap contains information from Statistics Canada, licensed under Open Government Licence – Canada, from R package *canadamaps* v2.0.0 (Cayen 2024).
