# Supplementary material for: The value of looking ahead: Comparing conventional and strategic Mountain Pine Beetle (Dendroctonus ponderosae) management policies in North America
Source: PLoS One. 2026 Jun 24;21(6):e0344860. doi: 10.1371/journal.pone.0344860 (PMC13293515; doi:10.1371/journal.pone.0344860)
Supplement: Attachment — Submitted filename: Review of Hudgins et al for PONE.docx [file pone.0344860.s004.docx]

Review of Hudgins et al for PLOS One

**The value of looking ahead: comparing conventional and strategic Mountain Pine Beetle (*Dendroctonus ponderosae*) management policies in North America.**

The manuscript tackles a very important topic: what strategies should be adopted to manage MPB outbreaks and more generally bark beetles? This is of primary importance for forest managers considering the potential of these species to transform ecosystems and generate major economic losses. The justification for the study is to minimise the economic cost of management while limiting MPB population growth and spread. The motivation is well stated and aligned with management challenges where resources are limited and decision-making is essential.

The authors compared one existing model under three management strategies: myopic, medium-term forecast and optimised long-term forecast. The myopic reactive management strategy uses only information from the current year infestations. At a given year, trees are controlled where infestations are detected. This scenario is similar to the approach used by the SMAC. The medium-term strategy involves forecasting the spread in the near future. The decision made each year is based on the forecast of the spread of the outbreak in the short term. At a given year, the controlled trees may not only be located in areas currently infested, but also in areas where the beetle is predicted to spread in the following year. The optimised long-term strategies use optimization to find the best management strategy over the longer time scale of the outbreak. This approach evaluates how decisions, such as tree removal, will affect the future beetle spread. At a given year, the controlled trees are not necessarily located where the infestations are recorded this year, but where beetles are predicted to spread over the entire period of the outbreak. This requires forecasting all combinations of growth and spread over multiple years under different tree-removal decisions within the available budget. This allows the optimisation to prioritise actions that have the greatest long-term impact.

The manuscript is generally clearly written, though some sections and the organization could be improved (see comments). The introduction to the problem and background information are outlined satisfactorily. The model is described, but the current structure makes it difficult to obtain a clear overview of the modelling process. The authors provide code to run the spread model, which appears to be based on Goodsman et al., 2024. However, this reference is not currently available online. This makes it difficult to assess how good the underlying model for spread is. Although the appendix and GitHub code are meant to provide enough information to reproduce the study results, it would greatly help if there were a flowchart diagram that describes the overall modelling process.

The results were interpreted in relation to management objectives, and the comparison of strategies provides useful insights for improving the cost-effectiveness of MPB and bark beetle outbreak response. The results are promising and support the idea that integrating forecasting and optimization can improve management efficiency. The discussion provides interpretations and opinions, explains the results and makes suggestions for management. However, it does not address the sensitivity analysis. A paragraph summarizing the key effects of parameter variation and their implications for interpretation would be beneficial to the reader.

The strength of the paper lies in the use of an optimisation approach which appears particularly well suited to this type of management problem. In addition, understanding whether the three management strategies produce similar outcomes and under which conditions the simplest and full models diverge or converge, is essential to align the modelling approach with management timelines.

The authors appear to miss much of the existing literature on applying optimization to control of invasive pests. Some of this can be found in Chapter 10 of Lewis et al. (2016). Of note is the work by Hastings and coauthors on using linear programming to stem the spread of *Spartina* in salt marshes.

**Comments**:

Even if we quickly understand the purpose of the study, the research question is not clearly stated as a question in the manuscript. It is recommended to give a clear formulation. There is a great deal of potential of using optimization to tackle these questions. Such optimization-based management do not appear to be well used in the bark beetle literature. However, the authors should discuss similar applications to other invasive species that already exist in the literature.

It is not entirely clear whether the manuscript aims to make a theoretical contribution or whether it is intended as an applied decision-support tool for managers. It appears to be more a proof of concept. The work represents a first step in applying optimization methods to questions on MPB management strategies. However, certain aspects of the manuscript would benefit from discussion to make it a fully usable tool for future management.

The authors rely on an existing spread model (Goodsman et al., 2024), with parameter values selected so that the model reproduces historical MPB infestation patterns in Alberta over recent decades (lines 273–279). As stated in the text, these values were chosen to reflect a typical unmanaged outbreak dynamics in western Canada. The model is initialised using the 2023 infestation map and used to forecast spread on a 11-year planning horizon from 2024 to 2034 under different management scenarios. As noted above, it is essential to have access to this paper in order to fully evaluate the model.

The formulation of the model via the 20 equations is not friendly for non-mathematicians. The set theory and logical symbols are not needed, and it would help greatly if each equation or inequality had a “word equivalent” written underneath using underbrackets so non-mathematicians could understand what the equations mean.

Table 1 could be improved. It is not clear why a semicolon is used when delineating the endpoints of a continuous interval. It is not clear what the “]” notation means for a left hand interval end. If one is to be consistent then the symbol should not be used to express the range, eg, B>0 would be replaced by (0,\infty) and so forth. It would be good to include the units as appropriate in the description. What is distance measured in? What about M? What about y_i? It would also help to know what the typical values are, and these could be included in the table as well, even though they appear in the text.

As a first step, do the authors initialize the model at the beginning of a known outbreak in the past, for example before the shift from endemic to epidemic conditions in Alberta, and then generate forecasts from this year onward? By doing so, the authors would have an idea of how good they are at predicting the growth and spread of the outbreak when confronted with data. Because with the current approach we don not know how accurate the model is to forecast infestations in the future and under varying conditions.

The manuscript also states that the parameter values were chosen so that the simulated spread area in the absence of management matched those of the model of Goodsman et al. (2024). It is not clear what this means from a statistical perspective. Are the parameters uniquely identifiable based only on this constraint? It would be helpful to have some further explanation here in the main text.

The equations 21 to 24 appear to be describing the model. If so, they should appear in the Methods, not the Results. Also, the number of decimal places that parameters are given appears to be very high (in some cases 10 decimal places). It is likely that these estimates are not nearly this precise. It may be that the methods preclude confidence interval estimations for parameters, but the number of decimal places seems to be rather misleading.

In terms of discussion, the parameter values do not consider that conditions are changing under climate change. For the model to be used for future management planning, it needs to account for key environmental variables that drive the growth and dispersal dynamics. At present, the model incorporates pine density, which is an essential driver of MPB dynamics. However, a handful of other environmental factors also play a major role in MPB outbreaks.

First, pine species matter. Although growth rates seem relatively similar between jack pine and lodgepole pine, dispersal dynamics is different across these hosts. The current model does not distinguish between spread in jack pine versus lodgepole pine forests. There is a recent recent paper on the topic:<https://doi.org/10.1002/ece3.72296>. This finding suggests that MPB exhibits reduced aggregation on jack pine and thus has a lower eruptive potential compared with lodgepole pine.

Second, outbreak transitions from endemic to epidemic phases are influenced by environmental conditions. For example, drought stress weakens host trees and reduces their defensive capacity, making it easier for beetles to overcome the Allee effect. Proxy variables of tree defense such as climate suitability index are important mechanistic predictors for outbreak initiation and growth. In contrast, cold temperatures, especially during late fall and early winter can substantially reduce beetle survival and hamper outbreak progression.

Third, long-dispersal is influenced by atmospheric conditions. MPB fly during warm, fair weather, conditions that can produce air inversions and upward convection currents that lift a small fraction of the population above the canopy (Safranyik & Carroll 2006). Therefore, wind direction is one the major determinants of long-distance dispersal, particularly given that MPB are relatively bad flyers.

An extension to the model would be to allow growth and dispersal equations to depend on those environmental variables. Incorporating them would likely affect management decisions. Incorporating these variables in the model increases the complexity of the optimization problem, especially for the full complexity model. It might be easier to implement it using the simple and reduced-complexity approach and see how much it changes the management decisions. While this adds complexity and uncertainty, the manuscript would benefit from discussing their impact on management strategies.

That being said, the authors also include a sensitivity analysis to examine how changes in parameter values affect spatial management priorities. This is useful but given the simplified treatment of environmental drivers in the model, it would help to discuss to what extent these sensitivity scenarios capture the uncertainty associated with omitted environmental drivers. The clarification would improve the interpretation of the resulting management priorities.

**Lines by lines comments**:

- line 80-84: suggest adding a slightly more nuanced version. While warming may synchronise emergence and reduce overwinter mortality, a higher degree of warming can also push development into less cold-hardy life stages (e.g. adults), increasing mortality risk. It is generally accepted that a certain degree of warming will have a positive impact on the MPB dynamic, but up to a certain point where warming can also be detrimental. For example, higher temperature does not necessarily mean faster growth if the energy surplus is invested in maintenance instead of growth. We do not know yet the full consequences of climate change on the MPB population dynamic.
- line 116: It might be worth looking at recent papers that evaluate the impact of control on MPB dynamics (see <https://doi.org/10.1111/1365-2664.70178> and <https://cdnsciencepub.com/doi/10.1139/cjfr-2018-0301> ).
- lines 155: There is a typo in the sentence. It currently says, “these factors makes can be challenging.” I believe you meant “these factors can be challenging.”
- line 546: In the tables, ensure consistency in naming the baseline scenario. Table 2 refers to it as base, whereas Tables 3 and 4 use “baseline scenario. Also, do not forget to define what is the baseline scenario.
- line 621: It is not easy to distinguish the colors as all the points are aggregated, I would try to remove the black outlines to see if this improves the visibility. Also, is the visibility better if you create 3 frequency bins instead of 2?
- line 782: heavy sentence can probably split into 2.
- line 801: There is a typo in the sentence. It currently says, "forecasting rare", did you mean forecasting rate?
- Line 906: The reference is not found online.
- References section: Several references are not formatted as the Vancouver style.

Reference

Lewis, M.A., Petrovskii, S., Potts, J. (2016) The Mathematics Behind Biological Invasions. Springer.
