# Supplementary material for: The value of looking ahead: Comparing conventional and strategic Mountain Pine Beetle (Dendroctonus ponderosae) management policies in North America
Source: PLoS One. 2026 Jun 24;21(6):e0344860. doi: 10.1371/journal.pone.0344860 (PMC13293515; doi:10.1371/journal.pone.0344860)
Supplement: Attachment — Submitted filename: plos_revisions.docx [file pone.0344860.s006.docx]

Journal Requirements:

When submitting your revision, we need you to address these additional requirements.

1. Please ensure that your manuscript meets PLOS ONE's style requirements, including those for file naming. The PLOS ONE style templates can be found at

<https://journals.plos.org/plosone/s/file?id=wjVg/PLOSOne_formatting_sample_main_body.pdf> and

[https://journals.plos.org/plosone/s/file?id=ba62/PLOSOne_formatting_sample_title_authors_affiliations.pdf](https://track.editorialmanager.com/CL0/https:%2F%2Fjournals.plos.org%2Fplosone%2Fs%2Ffile%3Fid=ba62%2FPLOSOne_formatting_sample_title_authors_affiliations.pdf/1/010f019b36621c22-374edee5-913e-451a-9983-987fe572f9e0-000000/_J968YxQEULTLiMxEPCQYIBQitG3WQ498a07_eWMGZU=242)

**Response:** This has been done.

2. Please note that PLOS One has specific guidelines on code sharing for submissions in which author-generated code underpins the findings in the manuscript. In these cases, we expect all author-generated code to be made available without restrictions upon publication of the work. Please review our guidelines at <https://journals.plos.org/plosone/s/materials-and-software-sharing#loc-sharing-code> and ensure that your code is shared in a way that follows best practice and facilitates reproducibility and reuse.

**Response:** We have ensured our code follows best practices as requested.

3. Please note that funding information should not appear in any section or other areas of your manuscript. We will only publish funding information present in the Funding Statement section of the online submission form. Please remove any funding-related text from the manuscript.

**Response:** Funding information was removed from the Acknowledgements.

4. Thank you for stating the following financial disclosure:

“NSERC Postdoctoral Fellowship awarded to EJH”

Please state what role the funders took in the study.  If the funders had no role, please state: "The funders had no role in study design, data collection and analysis, decision to publish, or preparation of the manuscript."

If this statement is not correct you must amend it as needed.

Please include this amended Role of Funder statement in your cover letter; we will change the online submission form on your behalf.

**Response:** The funders had no role in study design, data collection and analysis, decision to publish, or preparation of the manuscript. This has been mentioned in our cover letter.

5. Please expand the acronym “NSERC” (as indicated in your financial disclosure) so that it states the name of your funders in full.

This information should be included in your cover letter; we will change the online submission form on your behalf.

**Response:** This has been done.

6. Please note that your Data Availability Statement is currently missing the repository name and/or the DOI/accession number of each dataset OR a direct link to access each database. If your manuscript is accepted for publication, you will be asked to provide these details on a very short timeline. We therefore suggest that you provide this information now, though we will not hold up the peer review process if you are unable.

**Response:** We have created a DOI and added it to the Data Availability Statement (at <https://doi.org/10.5281/zenodo.18228184>).

7. We note that Figures 3,4, S4,S6,S8,S10,S12,S14,S16,S18,S20,S22,S24 and S26 in your submission contain map/satellite images which may be copyrighted. All PLOS content is published under the Creative Commons Attribution License (CC BY 4.0), which means that the manuscript, images, and Supporting Information files will be freely available online, and any third party is permitted to access, download, copy, distribute, and use these materials in any way, even commercially, with proper attribution. For these reasons, we cannot publish previously copyrighted maps or satellite images created using proprietary data, such as Google software (Google Maps, Street View, and Earth). For more information, see our copyright guidelines: <http://journals.plos.org/plosone/s/licenses-and-copyright>.

 We require you to either (1) present written permission from the copyright holder to publish these figures specifically under the CC BY 4.0 license, or (2) remove the figures from your submission:

 1. You may seek permission from the original copyright holder of Figures 3,4, S4,S6,S8,S10,S12,S14,S16,S18,S20,S22,S24 and S26 to publish the content specifically under the CC BY 4.0 license.

 We recommend that you contact the original copyright holder with the Content Permission Form (<http://journals.plos.org/plosone/s/file?id=7c09/content-permission-form.pdf>) and the following text:

“I request permission for the open-access journal PLOS ONE to publish XXX under the Creative Commons Attribution License (CCAL) CC BY 4.0 (<http://creativecommons.org/licenses/by/4.0/>). Please be aware that this license allows unrestricted use and distribution, even commercially, by third parties. Please reply and provide explicit written permission to publish XXX under a CC BY license and complete the attached form.”

 Please upload the completed Content Permission Form or other proof of granted permissions as an "Other" file with your submission.

 In the figure caption of the copyrighted figure, please include the following text: “Reprinted from [ref] under a CC BY license, with permission from [name of publisher], original copyright [original copyright year].”

 2. If you are unable to obtain permission from the original copyright holder to publish these figures under the CC BY 4.0 license or if the copyright holder’s requirements are incompatible with the CC BY 4.0 license, please either i) remove the figure or ii) supply a replacement figure that complies with the CC BY 4.0 license. Please check copyright information on all replacement figures and update the figure caption with source information. If applicable, please specify in the figure caption text when a figure is similar but not identical to the original image and is therefore for illustrative purposes only.

The following resources for replacing copyrighted map figures may be helpful:

 USGS National Map Viewer (public domain): <http://viewer.nationalmap.gov/viewer/>

The Gateway to Astronaut Photography of Earth (public domain): <http://eol.jsc.nasa.gov/sseop/clickmap/>

Maps at the CIA (public domain): <https://www.cia.gov/library/publications/the-world-factbook/index.html> and <https://www.cia.gov/library/publications/cia-maps-publications/index.html>

NASA Earth Observatory (public domain): <http://earthobservatory.nasa.gov/>

Landsat: <http://landsat.visibleearth.nasa.gov/>

USGS EROS (Earth Resources Observatory and Science (EROS) Center) (public domain): [http://eros.usgs.gov/#](http://eros.usgs.gov/)

Natural Earth (public domain): [http://www.naturalearthdata.com/](http://track.editorialmanager.com/CL0/http:%2F%2Fwww.naturalearthdata.com%2F/1/010f019b36621c22-374edee5-913e-451a-9983-987fe572f9e0-000000/AVaZtNcUIa2mzn3u7QE6vrErQrn4_kuKaPDnVkqJnw8=242)

**Response:** Our basemaps come from the R package *canadianmaps* which links directly to Statistics Canada Census boundaries, and which are licensed under a CC-BY license - https://www.statcan.gc.ca/en/terms-conditions/open-licence). We now note this and attribute this source in all relevant Figure captions.

8. We note you have included a table to which you do not refer in the text of your manuscript. Please ensure that you refer to Table 1 in your text; if accepted, production will need this reference to link the reader to the Table.

**Response:** This table has been moved to the supplement as Appendix 2 Table S1, and is now referred to in text (L405).

9. Please include captions for your Supporting Information files at the end of your manuscript, and update any in-text citations to match accordingly. Please see our Supporting Information guidelines for more information: <http://journals.plos.org/plosone/s/supporting-information>.

**Response:** This has been done.

10. If the reviewer comments include a recommendation to cite specific previously published works, please review and evaluate these publications to determine whether they are relevant and should be cited. There is no requirement to cite these works unless the editor has indicated otherwise. 

**Response:** We have attached the draft manuscript from Goodsman and colleagues for review by reviewers as well as two markdown documents describing the results we used, and have cited a selection of references within Chapter 10 of the textbook mentioned where relevant (See Reviewer 2 Responses).

Additional Editor Comments:

The reviewers’ comments were generally positive regarding the relevance of the study and the clarity of the writing. However, some points require clarification, particularly the need to state the research question more explicitly and to provide greater transparency regarding the choice and calibration of model parameters.

**Response:** We thank the editor for their positive comments and detail below how we have addressed their requests for clarification.

Reviewer #1: The manuscript titled “The value of looking ahead: comparing conventional and strategic Mountain Pine Beetle (Dendroctonus ponderosae) management policies in North America” is well-written with sufficient biological background and mathematical detail. The results of their optimization program have important implications for mountain pine beetle management. I have made some high-level comments below followed by more specific comments for sections and figures.
The main claims of this paper are that considering the impact of MPB spread and the impact of management decisions on future spread reduces the future MPB infested area. Considering that reducing the spread of MPB is a priority for the government of Alberta, these claims are significant for management of MPB. The study is also an important example of how optimization methods along with forecasting of management implications can be used to inform current management decisions. The manuscript is well organized in terms of logic and flow. The writing was accessible to me, a non-specialist in MPB.

**Response:** We thank the reviewer for their positive comments.

The level of mathematical detail given is sufficient for the study to be reproduced. However, Goodsman et al. 2024, which has been cited 5 times, including for the justification of key parameters, is not available through a Google Scholar search. I would strongly suggest making this information available, to make this work fully understandable and reproducible.

**Response:** This manuscript has not yet been submitted, so we could not make it available publicly, but have attached it to our revised submission for reviewers to use in their assessment along with two markdown documents describing the specific results we used here. We wish to clarify that Goodsman et al. was only used to derive an estimate of the effectiveness of management on spread rates in order to back-calculate spread rates in the absence of management. We now note this more explicitly in text (L204-212). Given this work is from conference proceedings and the associated manuscript is in preparation, we now also cite additional references on the effectiveness of management. We also note that spread dynamic assumptions were tested heavily in our sensitivity analysis.

Overall, I think this manuscript and work has potential and the authors should be encouraged to submit a revised version.

Some inline comments:
Line 36 – does “long-range” pest spread mean long distance or long-term (in terms of time)?

**Response:** We have changed this to longer-term (L54)

Line 328 – Why is it reasonable to not consider the impact of tree removal in the current year on future rates of MPB spread? Does that align with the current management framework?

**Response:** This sentence was meant to caveat that our single-period model was a slightly oversimplified case compared to true management actions, as even SMAC considered 1-3 years of future spread (not accounting for tree removal’s impact on that spread, but assessing future spread capacity of each cell). Current management does not account for the impact of tree removal on future MPB spread when assessing a site’s future spread capacity. We did this to contrast a completely myopic strategy (within a year) with one that examined much longer-term dynamics than 1-3 years (10+ years). While attempts to account for spread capacity e.g. drawing buffers around a cell) can account for near-term spread, they cannot account for longer term spread or the impact of management on spread, which requires more research effort on the computational side, which is not always available during operational management. We now explain this more clearly in text (L366-377)

Table 1 – w_it is a relative value. “Relative to carrying capacity” needs to stated.

**Response:** This has been changed here and throughout the methods section (note that Table 1 is now Appendix 2 Table S1)

Line 500 – Does each timestep correspond to a year?

**Response:** Yes – this has been noted (L483, Fig 1)

Line 546 - The sensitivity analysis is a great way to check the stability of the results, however, from the Tables 2 and 4, it is not clear what “key parameter changes” or “key differences across the sensitivity analyses” mean.

**Response:** This has been clarified as referring to any model parameter value (Table 2, L522) and large differences in the number of sites managed at different lags after detection (Table 4 legend)

Line 575 – The root mean squared error on 1982.5km^2 seems high. Can you justify why this level is okay?

**Response:** While this difference seems high, it corresponds to either over or underpredicting spread by 79.3 individual 25km^2^ cells on average over time, when there are a total of 7925 potentially invaded cells at any time, and this model was fit without any spatial parameters. We also note that the metric of fit is calculated based on a rough estimate of the additional spread expected in the absence of management. We detail below in response to Reviewer 2’s comments how this model is not meant to perfectly capture accurate MPB spread patterns, but rather examine the relative benefit of a more complex management strategy.

Discussion – It would be great to have a paragraph about trade-offs with the different models and a cost-benefit analysis. The discussion is otherwise well-written, accessible to wide audience and puts the results in the management perspective.

**Response:** The focus of this manuscript is to consider the benefit of varying management prioritisation strategies under a fixed budget. As such, all strategies have the same maximum budget (which we vary in our sensitivity analysis). Beyond management costs, we attempted to quantify the relative time cost of each of these models in terms of their solving times and computational skills required (now L529-531). The budget levels we considered were within the typical extent of surveillance efforts undertaken by the provinces involved, and given there are no plans to expand these budgets, a cost benefit analysis beyond this budget was considered out of scope.

The rationale behind our multiperiod model was to determine the benefit of accounting for the impact of management decisions on future long-term spread. Cost-benefit analysis would require the creation of marginal cost curves etc. with computed MPB impacts for each additional investment in model complexity trading off with management budgets. There is no straightforward way to quantify the investment into developing each of these model types by managers. Thinking only about management costs, and not model development costs, the optimisation models we show here are essentially a more complex cost-benefit analysis, because each optimisation run chooses truly optimal management locations for a given budget under a particular strategy. We think our model can be used as an input to a future CBA, but not vice versa.

We could have written our single period with spread model in a much more user-friendly R wrapper such as *prioritizr* if we didn’t intend to compare it with the more complex model implemented here, which government managers are likely to have the ability to implement with limited startup costs. We also note that the code written for this manuscript is made freely available and therefore has lower time costs for managers to use in prioritising spread, though we note it is written in a more complex mathematical notation. We now expand on these points in L753-759.

Comments about figures:

Figure 3 – The colours used for frequency are the same as the colours used for different models in the previous figure, which can be confusing. I would suggest using a different gradient colour scale. Moreover, I would suggest the cell border colour to be removed on made more transparent. The figure does not provide much information as it is currently and the black cell boundaries are overshadowing the information from the colours.

**Response:** We have changed the colour scheme of this figure (Now Fig 4) and removed the borders around grid cells, which we agree improves its clarity.

Figure 4 – This figure would be better if it was more zoomed in around the cells and if the transparency of the cell fill was increased.

**Response:** We chose the boundaries initially as we wanted to provide Alberta’s province-level context in this figure for people unfamiliar with the region. However, we see the benefit in better showing the pattern of invasion given the smaller grid cells. We gave thus edited the figure as shown. We did not feel the transparency needed to be increased in the new zoomed version as the grid cells do not overlap in this version, now Fig 5.

Reviewer #2: Review of Hudgins et al for PLOS One

The value of looking ahead: comparing conventional and strategic Mountain Pine Beetle (Dendroctonus ponderosae) management policies in North America.

The manuscript tackles a very important topic: what strategies should be adopted to manage MPB outbreaks and more generally bark beetles? This is of primary importance for forest managers considering the potential of these species to transform ecosystems and generate major economic losses. The justification for the study is to minimise the economic cost of management while limiting MPB population growth and spread. The motivation is well stated and aligned with management challenges where resources are limited and decision-making is essential.

The authors compared one existing model under three management strategies: myopic, medium-term forecast and optimised long-term forecast. The myopic reactive management strategy uses only information from the current year infestations. At a given year, trees are controlled where infestations are detected. This scenario is similar to the approach used by the SMAC. The medium-term strategy involves forecasting the spread in the near future. The decision made each year is based on the forecast of the spread of the outbreak in the short term. At a given year, the controlled trees may not only be located in areas currently infested, but also in areas where the beetle is predicted to spread in the following year. The optimised long-term strategies use optimization to find the best management strategy over the longer time scale of the outbreak. This approach evaluates how decisions, such as tree removal, will affect the future beetle spread. At a given year, the controlled trees are not necessarily located where the infestations are recorded this year, but where beetles are predicted to spread over the entire period of the outbreak. This requires forecasting all combinations of growth and spread over multiple years under different tree-removal decisions within the available budget. This allows the optimisation to prioritise actions that have the greatest long-term impact.

**Response:** We are glad to hear that the reviewer found these aspects of the study clear and important.

The manuscript is generally clearly written, though some sections and the organization could be improved (see comments). The introduction to the problem and background information are outlined satisfactorily. The model is described, but the current structure makes it difficult to obtain a clear overview of the modelling process. The authors provide code to run the spread model, which appears to be based on Goodsman et al., 2024. However, this reference is not currently available online. This makes it difficult to assess how good the underlying model for spread is. Although the appendix and GitHub code are meant to provide enough information to reproduce the study results, it would greatly help if there were a flowchart diagram that describes the overall modelling process.

**Response:** As mentioned above, we now provide the unpublished manuscript upon which our spread model is roughly based. We note that we did not aim to reproduce this more complex model’s small scale dynamics exactly, rather we used it only to offset past distributional records of MPB to model historical spread in the absence of management. Thus, our code and equations should suffice to understand model structure. We now include a flowchart diagram of our workflow for easier comprehension of all steps (now Fig 1).

The results were interpreted in relation to management objectives, and the comparison of strategies provides useful insights for improving the cost-effectiveness of MPB and bark beetle outbreak response. The results are promising and support the idea that integrating forecasting and optimization can improve management efficiency. The discussion provides interpretations and opinions, explains the results and makes suggestions for management. However, it does not address the sensitivity analysis. A paragraph summarizing the key effects of parameter variation and their implications for interpretation would be beneficial to the reader.

The strength of the paper lies in the use of an optimisation approach which appears particularly well suited to this type of management problem. In addition, understanding whether the three management strategies produce similar outcomes and under which conditions the simplest and full models diverge or converge, is essential to align the modelling approach with management timelines.

**Response:** We thank the reviewer for their positive comments on several manuscript sections. We have now added a discussion section that interprets the sensitivity analysis (L679-691)

The authors appear to miss much of the existing literature on applying optimization to control of invasive pests. Some of this can be found in Chapter 10 of Lewis et al. (2016). Of note is the work by Hastings and coauthors on using linear programming to stem the spread of Spartina in salt marshes.

**Response**: We thank the reviewer for calling to attention this important body of research. We did not aim to target this manuscript at optimisation experts, so we did not initially include extensive citation of optimal control literature. However, we agree that failing to mention optimal control altogether could overstate the novelty of this work. We have thus added some citations from this Chapter to L153-161.

Comments:

Even if we quickly understand the purpose of the study, the research question is not clearly stated as a question in the manuscript. It is recommended to give a clear formulation. There is a great deal of potential of using optimization to tackle these questions. Such optimization-based management do not appear to be well used in the bark beetle literature. However, the authors should discuss similar applications to other invasive species that already exist in the literature.

**Response:** We have now included a statement of the research question directly in the manuscript introduction (L179-182). As mentioned above, we have added other citations from optimal control.

It is not entirely clear whether the manuscript aims to make a theoretical contribution or whether it is intended as an applied decision-support tool for managers. It appears to be more a proof of concept. The work represents a first step in applying optimization methods to questions on MPB management strategies. However, certain aspects of the manuscript would benefit from discussion to make it a fully usable tool for future management.

**Response:** This is an important point of clarification. We aimed to make a more theoretical contribution in this work, rather than aiming to be immediately useable to managers in its current form. We agree that this work is the first step in motivating managers to account for long term spread when prioritising management locations for bark beetles. As such, our most complex model is likely out of scope for use in on-the-ground management, which is why we checked how much benefit there was in only modelling spread without incorporating the impact of management on this spread (Now explained on L183-186). We note the decreased research effort required in using R wrappers such as *prioritizr* in our current manuscript relative to our more complex form for this type of model. Future work could implement our prioritisation based on future spread in an R wrapper model for more direct uptake by managers. We now express this more explicitly on L753-759.

The authors rely on an existing spread model (Goodsman et al., 2024), with parameter values selected so that the model reproduces historical MPB infestation patterns in Alberta over recent decades (lines 273–279). As stated in the text, these values were chosen to reflect a typical unmanaged outbreak dynamics in western Canada. The model is initialised using the 2023 infestation map and used to forecast spread on a 11-year planning horizon from 2024 to 2034 under different management scenarios. As noted above, it is essential to have access to this paper in order to fully evaluate the model.

**Response:** We have now provided access to the Goodsman et al. manuscript.

The formulation of the model via the 20 equations is not friendly for non-mathematicians. The set theory and logical symbols are not needed, and it would help greatly if each equation or inequality had a “word equivalent” written underneath using underbrackets so non-mathematicians could understand what the equations mean.

**Response:** We have moved the majority of model equations to a supplement and kept only key equations in the main text in a simplified format (10 total equations with substantially reduced symbology), with sentences between each equation to explain the purpose of each.

Table 1 could be improved. It is not clear why a semicolon is used when delineating the endpoints of a continuous interval. It is not clear what the “]” notation means for a left hand interval end. If one is to be consistent then the symbol should not be used to express the range, eg, B>0 would be replaced by (0,\infty) and so forth. It would be good to include the units as appropriate in the description. What is distance measured in? What about M? What about y_i? It would also help to know what the typical values are, and these could be included in the table as well, even though they appear in the text.

**Response:** We have now moved this Table to the appendix in line with the reviewer’s suggestion to simplify all equations in the main text. Within the table in the appendix, we have added units and made range notation consistent (Appendix 2 Table S1). We note that many model components are relative to carrying capacity costs and densities, so they do not have units.

As a first step, do the authors initialize the model at the beginning of a known outbreak in the past, for example before the shift from endemic to epidemic conditions in Alberta, and then generate forecasts from this year onward? By doing so, the authors would have an idea of how good they are at predicting the growth and spread of the outbreak when confronted with data. Because with the current approach we don not know how accurate the model is to forecast infestations in the future and under varying conditions.

**Response:** Yes – we initialised the spread model using historical invasion data, offset by an empirically derived management efficiency term to account for the fact that past invasions were subject to management effort. This is how our metrics of fit were calculated. We also set our population dynamics parameters to roughly account for historical invasion dynamics in terms of shifts from endemic to epidemic phases as explained on L292-303. We did not aim to exactly predict historical spread extent within the optimisation itself, which is initialised at the present year, as we had already fit the spread model using this method. However, we note that the optimisation model reduces the extent of spread by roughly 1/3 under the myopic strategy, which matches historical management effectiveness. We have explained this more clearly on L

The manuscript also states that the parameter values were chosen so that the simulated spread area in the absence of management matched those of the model of Goodsman et al. (2024). It is not clear what this means from a statistical perspective. Are the parameters uniquely identifiable based only on this constraint? It would be helpful to have some further explanation here in the main text.

**Response:** Our model formulation is much more simplified compared to that of Goodsman et al (2024). Thus, we did not attempt to fit the same set of parameters. We fit only the population growth and dispersal kernel function such that the extent of spread over time was as close as possible to observed spread, offset by the effectiveness of management on slowing MPB spread estimated by Goodsman et al.’s model. We now explain this more clearly on L228-233.

The equations 21 to 24 appear to be describing the model. If so, they should appear in the Methods, not the Results. Also, the number of decimal places that parameters are given appears to be very high (in some cases 10 decimal places). It is likely that these estimates are not nearly this precise. It may be that the methods preclude confidence interval estimations for parameters, but the number of decimal places seems to be rather misleading.

**Response:** We had originally included these equations in the results because we saw the fitting of the spread model as a methodological step with results. We have now simplified all equations within the main text and moved them into the methods. We also now report all parameter estimates to 2 decimal places.

In terms of discussion, the parameter values do not consider that conditions are changing under climate change. For the model to be used for future management planning, it needs to account for key environmental variables that drive the growth and dispersal dynamics. At present, the model incorporates pine density, which is an essential driver of MPB dynamics. However, a handful of other environmental factors also play a major role in MPB outbreaks.

**Response:** We did not intend for our model to capture longer term pine dynamics under climate change, as our time window for optimisation is only a decade. We agree that future implementations of the single period plus spread model could include a more complex spread and MPB population growth component that accounts for climate, such as in the references mentioned below. (See the next three responses).

First, pine species matter. Although growth rates seem relatively similar between jack pine and lodgepole pine, dispersal dynamics is different across these hosts. The current model does not distinguish between spread in jack pine versus lodgepole pine forests. There is a recent recent paper on the topic: <https://doi.org/10.1002/ece3.72296>. This finding suggests that MPB exhibits reduced aggregation on jack pine and thus has a lower eruptive potential compared with lodgepole pine.

**Response:** We acknowledge that MPB dynamics vary according to pine species**.** When the SMAC began, and for much of the early MPB invasion, the assumption was that MPB would perform as well or better in jack pine because some of Cerezke’s work showed it would, as well they seemed to perform relatively similar in hybrid jack-lodgepole forests. So, for the purposes of management, jack and lodgepole were treated the same by SMAC. Only recently has MPB performance in jack pine been shown to be lower. The region of the MPB distribution we intended to model is not the area dominated by solely jack pine. As such, we feel this underlying assumption for the period of the model is valid. We do not believe that accounting for pine species would qualitatively change the best model type for management, as Alberta would still remove an infested jack pine and they would not change survey strategy based on species density (or at least they do not appear to have historically). We feel that accounting for species differences would be an especially important extension to the work if the model is to be used further east. We now mention these points and reference the associated paper on (L773-784).

Second, outbreak transitions from endemic to epidemic phases are influenced by environmental conditions. For example, drought stress weakens host trees and reduces their defensive capacity, making it easier for beetles to overcome the Allee effect. Proxy variables of tree defense such as climate suitability index are important mechanistic predictors for outbreak initiation and growth. In contrast, cold temperatures, especially during late fall and early winter can substantially reduce beetle survival and hamper outbreak progression.

**Response:** We have assumed consistent climate dynamics over the region and time period simulated. This is a simplification we think is defensible given the decade-long time window we have examined, but could be extended for longer term management plans, or for particularly steeply changing regions in terms of drought stress. However, very little data exists on historical monitoring of overwintering mortality, making this type of monitoring important for future modelling efforts. We now discuss this on L785-797.

Third, long-dispersal is influenced by atmospheric conditions. MPB fly during warm, fair weather, conditions that can produce air inversions and upward convection currents that lift a small fraction of the population above the canopy (Safranyik & Carroll 2006). Therefore, wind direction is one the major determinants of long-distance dispersal, particularly given that MPB are relatively bad flyers.

**Response:** We agree that the failure to account for directional spread beyond pine density is a limitation of this model. This additional complexity is present within Goodsman et al. (2024) and could be incorporated in future work in a single-period-plus-spread model (see below). It is quite complex to model the overlap between wind direction and rare, favourable conditions for long distance dispersal – likely requiring atmospheric modelling subject to very large uncertainty in future weather conditions. This would blow up the complexity of our optimisation, which is made feasible by a single dispersal equation, and even so requires substantial HPC capacity. We are unaware of existing models that predict these events effectively. We thus felt this level of complexity was outside of model scope. Our choice of isotropic kernel is meant to depict longer term, largescale average dispersal effects. We now explain this on L798-804.

An extension to the model would be to allow growth and dispersal equations to depend on those environmental variables. Incorporating them would likely affect management decisions. Incorporating these variables in the model increases the complexity of the optimization problem, especially for the full complexity model. It might be easier to implement it using the simple and reduced-complexity approach and see how much it changes the management decisions. While this adds complexity and uncertainty, the manuscript would benefit from discussing their impact on management strategies.

**Response:** Given the more theoretical aim of this work over a short time period, we feel that introducing this level of complexity is out of scope for this manuscript. Nevertheless, we discuss how these additions could be part of subsequent work (likely using advances in Goodsman et al. (2024) and other suggested citations) (L798-804). The proposed model can easily incorporate spatially variable environmental conditions – all variables (growth, minimum density on arrival etc) are indexed by site and can be made site specific (as we do in the case of pine density). This could be an exciting avenue of future research.

That being said, the authors also include a sensitivity analysis to examine how changes in parameter values affect spatial management priorities. This is useful but given the simplified treatment of environmental drivers in the model, it would help to discuss to what extent these sensitivity scenarios capture the uncertainty associated with omitted environmental drivers. The clarification would improve the interpretation of the resulting management priorities.

**Response:** We thank the reviewer for noting our examination of major drivers of qualitative change in management performance via our sensitivity analysis. We note that these substantial changes to model parameters showed limited qualitative differences in terms of management strategy. However, we agree that our sensitivity analysis focusses only on MPB population and management dynamic components, rather than environmental components that change dynamics spatially. We agree that there are plenty of other factors that could impact MPB spread. We now clarify these gaps that were not explored in our sensitivity analysis (L773,L813)

Lines by lines comments:
• line 80-84: suggest adding a slightly more nuanced version. While warming may synchronise emergence and reduce overwinter mortality, a higher degree of warming can also push development into less cold-hardy life stages (e.g. adults), increasing mortality risk. It is generally accepted that a certain degree of warming will have a positive impact on the MPB dynamic, but up to a certain point where warming can also be detrimental. For example, higher temperature does not necessarily mean faster growth if the energy surplus is invested in maintenance instead of growth. We do not know yet the full consequences of climate change on the MPB population dynamic.
**Response:** We thank the reviewer for this added nuance. Given our assumption of constant climate in our model, we moved any mention of climate change this to our limitations section (L785-797)

• line 116: It might be worth looking at recent papers that evaluate the impact of control on MPB dynamics (see <https://doi.org/10.1111/1365-2664.70178> and <https://cdnsciencepub.com/doi/10.1139/cjfr-2018-0301> ).
**Response:** These references are now mentioned on L123-126

• lines 155: There is a typo in the sentence. It currently says, “these factors makes can be challenging.” I believe you meant “these factors can be challenging.”
**Response:** This has been changed (L165)

• line 546: In the tables, ensure consistency in naming the baseline scenario. Table 2 refers to it as base, whereas Tables 3 and 4 use “baseline scenario. Also, do not forget to define what is the baseline scenario.
**Response:** This has been changed and defined in the tables and L531

• line 621: It is not easy to distinguish the colors as all the points are aggregated, I would try to remove the black outlines to see if this improves the visibility. Also, is the visibility better if you create 3 frequency bins instead of 2?
**Response:** We removed the black outlines as suggested by Reviewer 1 in this figure. We had 5 frequency bins, not 2, so we believe the Reviewer refers to the increase in frequency between bins. We have made a new version of this figure with jumps of 3.

• line 782: heavy sentence can probably split into 2.
**Response:** We now use two sentences here (L766)

• line 801: There is a typo in the sentence. It currently says, "forecasting rare", did you mean forecasting rate?
**Response:** This was not a typo – the phrase “Given the challenge of forecasting rare, long distance spread events” is grammatically correct

• Line 906: The reference is not found online.
**Response:** We included this reference as the associated manuscript has not been published, but the conference proceedings are available online here: https://entomology2024.eventscribe.net/biography.asp?pfp=Speakers, and have uploaded the unpublished

• References section: Several references are not formatted as the Vancouver style.

**Response:** This has been corrected (see also editorial comments above).

Reference

Lewis, M.A., Petrovskii, S., Potts, J. (2016) The Mathematics Behind Biological Invasions. Springer.
